# Marine phytoplankton downregulate core photosynthesis and carbon storage genes upon rapid mixed layer shallowing

**DOI:** 10.1038/s41396-023-01416-x

**Published:** 2023-05-08

**Authors:** Ben P. Diaz, Ehud Zelzion, Kimberly Halsey, Peter Gaube, Michael Behrenfeld, Kay D. Bidle

**Affiliations:** 1 Department of Marine and Coastal Science, Rutgers University, New Brunswick, NJ 08901 USA; 2 Office of Advanced Research Computing, Rutgers University, Piscataway, NJ 08854 USA; 3 Department of Microbiology, Oregon State University, Corvallis, OR 97331 USA; 4 Applied Physics Laboratory, University of Washington, Seattle, WA 98105 USA; 5 Department of Botany and Plant Pathology, Oregon State University, Corvallis, OR 97331 USA; 6 Present Address: Biotechnology & Bioengineering, Sandia National Laboratories, 7011 East Avenue, Livermore, CA 94550 USA

**Keywords:** Water microbiology, Microbial ecology, Microbial communities

## Abstract

Marine phytoplankton are a diverse group of photoautotrophic organisms and key mediators in the global carbon cycle. Phytoplankton physiology and biomass accumulation are closely tied to mixed layer depth, but the intracellular metabolic pathways activated in response to changes in mixed layer depth remain less explored. Here, metatranscriptomics was used to characterize the phytoplankton community response to a mixed layer shallowing (from 233 to 5 m) over the course of two days during the late spring in the Northwest Atlantic. Most phytoplankton genera downregulated core photosynthesis, carbon storage, and carbon fixation genes as the system transitioned from a deep to a shallow mixed layer and shifted towards catabolism of stored carbon supportive of rapid cell growth. In contrast, phytoplankton genera exhibited divergent transcriptional patterns for photosystem light harvesting complex genes during this transition. Active virus infection, taken as the ratio of virus to host transcripts, increased in the Bacillariophyta (diatom) phylum and decreased in the Chlorophyta (green algae) phylum upon mixed layer shallowing. A conceptual model is proposed to provide ecophysiological context for our findings, in which integrated light limitation and lower division rates during transient deep mixing are hypothesized to disrupt resource-driven, oscillating transcript levels related to photosynthesis, carbon fixation, and carbon storage. Our findings highlight shared and unique transcriptional response strategies within phytoplankton communities acclimating to the dynamic light environment associated with transient deep mixing and shallowing events during the annual North Atlantic bloom.

## Introduction

Phytoplankton are unicellular photoautotrophs that support marine food webs across ocean regions through seasonal accumulation in the surface mixed layer (ML), the uppermost region in the ocean that is homogenized by turbulent mixing or convection. Seasonal phytoplankton blooms are intimately linked to mixed layer depth (MLD) [1, 2] where increasing sunlight and warmth in the spring cause the ML to shallow and phytoplankton to be exposed to higher daily irradiance than during the winter, resulting in high surface biomass accumulation [1]. In the North Atlantic, annual cycles in phytoplankton biomass reflect temporal changes in both prokaryotic and eukaryotic species, but during the spring bloom climax it is the eukaryotic species that dominate biomass. This North Atlantic event is one of the largest blooms in the global ocean, and its annual cycling was recently investigated during the North Atlantic Aerosol and Marine Ecosystem Study (NAAMES) [3–9]. The NAAMES study emphasized links between the physical environment (e.g., MLD changes) and the community composition and ecophysiology of bloom-dominating eukaryotic phytoplankton [4, 5, 7, 10, 11].

Previous fieldwork has characterized bulk eukaryotic phytoplankton community responses in the North Atlantic to changes in MLD on time scales of days to months [4, 8–10, 12]. In spring, storms episodically disturb surface waters and deepen the MLD below the euphotic zone, resulting in replenishment of the ML with nutrients from below and phytoplankton growth that is transiently light limited [10]. We recently showed that, upon shallowing, phytoplankton communities exhibit signatures of pervasive oxidative stress (oxidized membrane lipids, intracellular ROS, and transparent exopolymer particle production) and positive viral production when biomass is accumulating in late spring [4]. Following the spring climax phase, the ML becomes more strongly and stably stratified in the summer and autumn. This prolonged stratification leads to nutrient deprivation, decreases in phytoplankton concentrations, negative accumulation rates, and signatures of compromised membranes, death-related protease activity, and virus production, all associated with increased phytoplankton removal mechanisms such as increased predator/virus concentrations and programmed cell death [4].

A growing body of work has also identified metabolic responses to dynamic light availability in lab cultures for diatoms and chlorophytes, two common phytoplankton taxa found in the North Atlantic [5, 7]. When exposed to higher irradiance than that to which they are acclimated, these same taxa upregulate specific light harvesting chlorophyll-protein complexes (LHCs) [13–15], such as LHCX which participates in photoprotection [16, 17], as well as accessory pigments [10], and antioxidants [14]. Diatoms and chlorophytes conversely downregulate core photosystem genes [14, 18–20] and other LHC [13–15, 21]. Studies are still discerning the intracellular arrangement [22] and function of many LHC proteins in diatoms, which seem to be up and downregulated within the same class in response to high light [13, 14]. The transition from low to high light is also associated with an increase in biomass accumulation rate in both diatoms and chlorophytes [15, 23, 24].

An important factor to consider when interpreting in situ transcriptional states of phytoplankton is the effect of the diel irradiance cycle on photosynthesis and cell division. In field studies of stable MLs that are sufficiently shallow to reside within the euphotic zone, eukaryotic and prokaryotic phytoplankton communities have been shown to upregulate transcripts coding for core photosystem elements (photosystem II subunits and ATPase), a subset of LHC’s, and carbon fixation (Calvin cycle) starting in the night. These gene sets reach their maximum transcriptional levels hours following sunrise [25–31]. Conversely, transcription related to oxidative phosphorylation and lipid storage molecules (triacylglycerol) reach their highest level as the sun sets and decrease through the night [25, 27, 31, 32], supportive of cell division. In lab cultures of model diatoms grown under 12 h photoperiod cycles, carbon storage (fatty acid) biosynthesis transcription is upregulated at the beginning of the photoperiod, while cell division and DNA replication genes are downregulated [33]. These patterns are reversed by the end of the photoperiod [33], reflecting a shift towards carbon energy storage utilization of stored carbon as darkness begins. These findings imply that phytoplankton will decrease the transcriptional commitment to core photosystem components and begin to store carbon in preparation for cell division when their daily integrated irradiance is at least as high as the irradiance to which they are acclimated.

Here, we identify specific intracellular pathways, genes, and viral infection states associated with phytoplankton communities during a rapid (~2 d) ML shallowing event at a NAAMES station during the Spring climax phase. Resident phytoplankton had been entrained in a MLD of 233 m, which was remnant of deep winter mixing and the passing of a large storm system. The MLD was 180 m deeper than the euphotic zone (51 m), taken as the depth of 1% surface irradiance. The ML subsequently shallowed to 5 m, with the median light level corresponding to 40% of surface irradiance. Previous characterization of the bulk phytoplankton community response to rapid shallowing at this same station contextualizes our study in terms of accumulation [4, 9], photoacclimation [10], predators/viruses [4, 8], storage lipids [4], and oxidative stress [4]. Cell concentrations of eukaryotic phytoplankton (1–20 µm diameter) increased in the surface waters of the shallow ML compared to that of the deep ML [4, 10], indicating that this shallowing event induced cells to divide rapidly. Accumulation was accompanied by photoacclimation processes, including increases in accessory pigments and photosystem cross sectional area, decreases in the number of photosystem II (PSII) reaction centers per phytoplankton biomass and total chlorophyll fluorescence per cell of eukaryotic phytoplankton [10]. Intracellular lipid storage increased in the shallow mixed layer [4], while oxidative stress biomarkers such as ROS, oxidized membrane lipids, and transparent exopolymers per cell did not show a consistent pattern [4], suggestive of a mixed oxidative stress response in the community. At the same time, total predators (grazers and viruses) increased, while free virus:cell ratios decreased [4, 8], allowing for a temporary decoupling of phytoplankton growth and predator abundance.

We used these extensive observations of community parameters at this station, combined with the aforementioned understanding of how low light acclimated phytoplankton transition to high light exposure, to construct an interpretive framework for metatranscriptomic responses of the resident eukaryotic phytoplankton to rapid ML shallowing. We hypothesized that the transition of phytoplankton from light limited (deep ML) to light-saturated (shallow ML) states would downregulate core photosystem genes and Calvin cycle genes, but upregulate genes encoding for carotenoids, oxidant-scavenging proteins, carbon storage (triacylglycerols or complex carbohydrates), and specific photosynthetic LHC. Given rapid phytoplankton accumulation and the low bulk virus:cell ratio associated with recently shallowed ML communities at this station [4], we further hypothesized that active viral replication within resident cells would decrease compared to deep ML communities, removing viral pressure and allowing cells to accumulate more rapidly.

## Results/Discussion

### Rapid mixed layer shallowing in the North Atlantic

**Fig. 1:**
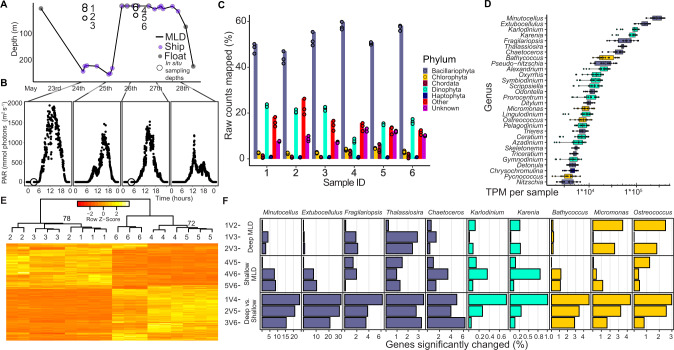
Phytoplankton community taxonomy and conserved transcriptional response to rapid stratification. **A** Sampling profile in relation to mixed layer depth (MLD). Open circles (1–6) represent depths and times sampled for metatranscriptome analyses. They correspond to a suite of previously published physiological markers [4]. Closed circles and line graph represent mixed layer depth, based on ship and optical float profilers (color coded; see key). **B** Sea level photosynthetically active radiation derived from shipboard measurements, aligned with the time in (**A**). Each data point in the PAR time series is the daily integrated broadband irradiance across the visible wavelengths (400–700 nm). **C** Percent of raw RNA reads mapped to taxa of interest and include prominent photoautotrophic phyla [Bacillariophyta (diatoms), Chlorophyta (green algae), Dinophyta (dinoflagellates), and Haptophyta (haptophytes)]. Colored circles indicate triplicates. **D** Top 20 represented genera based on summed TPM per sample. Each colored circle represents one triplicate from samples 1–6 in (**A**). Box plots represent median ±1 quartile. **E** Transcriptional response of top genera from Bacillariophyta (x5), Dinophyta (x2), and Chlorophyta (x3) phyla. Each row represents a unique gene that was determined to be differentially expressed (adjusted *p* value < 0.001, log_2_ fold change > |1|). Each column represents a sample triplicate from (**A**). Z scores, row order, and column grouping are based on a correlation matrix constructed from variance stabilized transformation of gene expression, using pairwise complete observations and Pearson coefficients. Nodes are only labeled if they were under 100 bootstraps (*n* = 1000, average method, correlation distance matrix, pairwise complete observations.). **F** Percentage of significantly changed genes by genus (adjusted *p* value <0.001, log_2_ fold change > |1|). “1V2”, “1V3”, “2V3”, etc. refer to sample number comparisons in (**A**).

We observed a rapid stratification event during the Climax phase of the annual phytoplankton biomass bloom cycle in the North Atlantic in late spring (24–26 May 2016) [3, 4, 8, 10]. At the time of site occupation (Station 4, NAAMES II [3]), phytoplankton biomass was already high throughout the North Atlantic compared to other seasonal phases and continued to positively accumulate [4, 9]. Phytoplankton populations in this seasonal bloom phase routinely experience episodic ML deepening and subsequent stratification associated with passing storms (Supplementary Fig. 1 and Supplementary Table 1) because the upper ML is still only weakly differentiated from deeper water [4, 34, 35]. Upon station arrival, the water column was deeply mixed, as determined by density gradients based on salinity and temperature (Supplementary Fig. 2B, C and Fig. 1A). Chlorophyll A concentrations were also uniformly distributed, suggesting an actively mixed or very recently mixed layer (Supplementary Fig. 2H, Supplementary Table 2). Case studies of this station revealed that we sampled within the core of an anticyclonic eddy on 24 May and were within the periphery of this eddy on 26 May [36, 37]. Our sampling strategy was to capture the transcriptional responses of a phytoplankton community to changes in MLD (and associated light levels) within this anticyclonic eddy using a nearby optical profiling float for guidance (Supplementary Fig. 1, “metbio003d” [3, 4, 10]).

We estimate that water was deeply mixed at least 2 days prior to arrival based on the optical profiling float, which was ~61 km away from our station occupation. (Fig. 1A). The MLD during station occupation extended from the surface to 233 m throughout 24 May and shoaled to 5 m by ~15:00 h on 25 May, exposing phytoplankton to higher daily irradiance until PAR decreased below 10 µmol photons m^2^ s^−1^ at ~22:00 h (Fig. 1A, B). This shallow MLD persisted throughout the remaining sampling period (Fig. 1A). Rapid ML shallowing and deepening on similar spatial and temporal scales as those observed during the current study (>100 m MLD to <25 m MLD within 72 h) can occur several times per year in some regions of the Northwest Atlantic (Supplementary Table 1, Supplementary Fig. 1). These rapid shallowing and deepening events happen most frequently from November through May in this region (Supplementary Fig. 1).

Integrated surface PAR levels were similar on the days prior to sampling days (May 23rd and May 25th, Supplementary Fig. 2A); these cumulative light levels would be expected to impact phytoplankton community transcription in predawn-collected cells. During occupation, integrated surface PAR slightly decreased (Fig. 1B). Modest macronutrient drawdown was observed in the shallow ML (Supplementary Fig. 2D–G, Supplementary Table 2) but not to growth-limiting levels. This modest nutrient drawdown was accompanied by increases in chlorophyll A and backscatter [10] within the shallow ML (Supplementary Fig. 2H), both proxies for phytoplankton biomass. The ratio of photosynthetic pigments to all pigments decreased (Supplementary Fig. 2I and Supplementary Table 2), suggesting a community-wide shift towards increased photoprotection [10].

### Community-wide, phytoplankton transcriptome response

We analyzed the eukaryotic phytoplankton community metatranscriptome to assess intracellular responses of phytoplankton to the stratification event. The taxonomic composition of cellular polyadenylated mRNA (hereafter referred as mRNA) within the euphotic zone was similar between the two predawn sampling days (see Methods; Fig. 1C). The top three representative unicellular phototrophic taxa were Bacillariophyta (diatoms), Dinophyta (dinoflagellates), and Chlorophyta (green algae or chlorophytes) (Fig. 1C). The most abundant diatom mRNA in all samples was from *Minutocellus*, a centric diatom that can form chains, followed by *Extobocellulus* and *Fragilariopsis* (Fig. 1D). The most represented Chlorophytes were *Micromonas*, *Bathycoccus*, and *Ostreococcus* genera (Fig. 1D), which generally agrees with 16S rRNA sequencing of the same community [11]. The mRNA taxonomic distribution may not reflect biomass abundance, as eukaryotic phytoplankton cell volume [38], RNA content per cell [39, 40], and genome size [41] can vary by orders of magnitude. Although dinoflagellates contributed a sizable proportion of community mRNA, they showed a relatively low percentage of genes differentially expressed within our two-day sampling window in response to stratification (Fig. 1F). This may reflect their well-documented muted transcriptional responses in favor of post translational regulation [42–44]. In addition, they are known to display mixotrophic transcriptional strategies in ocean regions with higher net primary productivity [45], which complicates interpretive analyses from a photoacclimation perspective. We therefore focused our attention on the responses to ML shallowing related to photosynthesis, carbon fixation, carbon storage and infection state within the autotrophic diatom and chlorophyte taxa.

The global transcriptional profiles of the most abundant phytoplankton genera were very closely tied to MLD (Fig. 1E, F). We compared transcripts between different light levels and MLDs and found a higher percentage of differentially expressed genes between different MLDs and the same light level compared to different light levels within the same MLD (Fig. 1E, F). The two most abundant genera, *Minutocellus* and *Extobocellulus*, had the largest percentage of differentially expressed genes in between the deep and shallow MLs (Fig. 1F), changing 20–30% of the detected transcripts. This was about one order of magnitude more than other diatoms and chlorophytes, and around two orders of magnitude more than dinoflagellate genera (Fig. 1F). In contrast, transcriptomes contained fewer differentially expressed genes between different light levels for most genera within the deep ML, (Fig. 1E, F) indicative of active mixing. Moderate differential gene expression was detected among samples collected from above and below the stratified ML, reflective of the physical barrier (pycnocline) to mixing. (Fig. 1E, F).

Given the similarities in phytoplankton and bacterial community compositions to other data collected (Fig. 1C) at the same station on 24 May and 26 May [7, 10, 46], along with the aforementioned physical data supporting sampling within the anticyclonic eddy [36, 37], the observed differential gene expression patterns most likely represent responses of the same core community members to water column stratification associated changes in irradiance. The idea that we sampled the same core community over our station occupation is further supported by geochemical measurements. Dimethylsulfide, a chemical that is released by marine algal and bacterial communities [47, 48], gradually increased as phytoplankton biomass increased at this station [49]; arguing production originating from the same microbial community in response to stratification. Due to the rapid timescale of ML shallowing observed, we acknowledge the possibility that warmer water may have laterally displaced the colder deeply mixed water within the anticyclonic eddy that we were sampling [36, 37] and contributed to shallow ML water on 26 May. If such a lateral transduction had occurred, the transcriptional responses may not represent the exact timescale these organisms use to respond to stratification. Nonetheless, our data reveal general subcellular phytoplankton responses to changes in light regimes that are inherently associated with dynamic MLDs.

### A variety of light harvesting transcriptional patterns

**Fig. 2:**
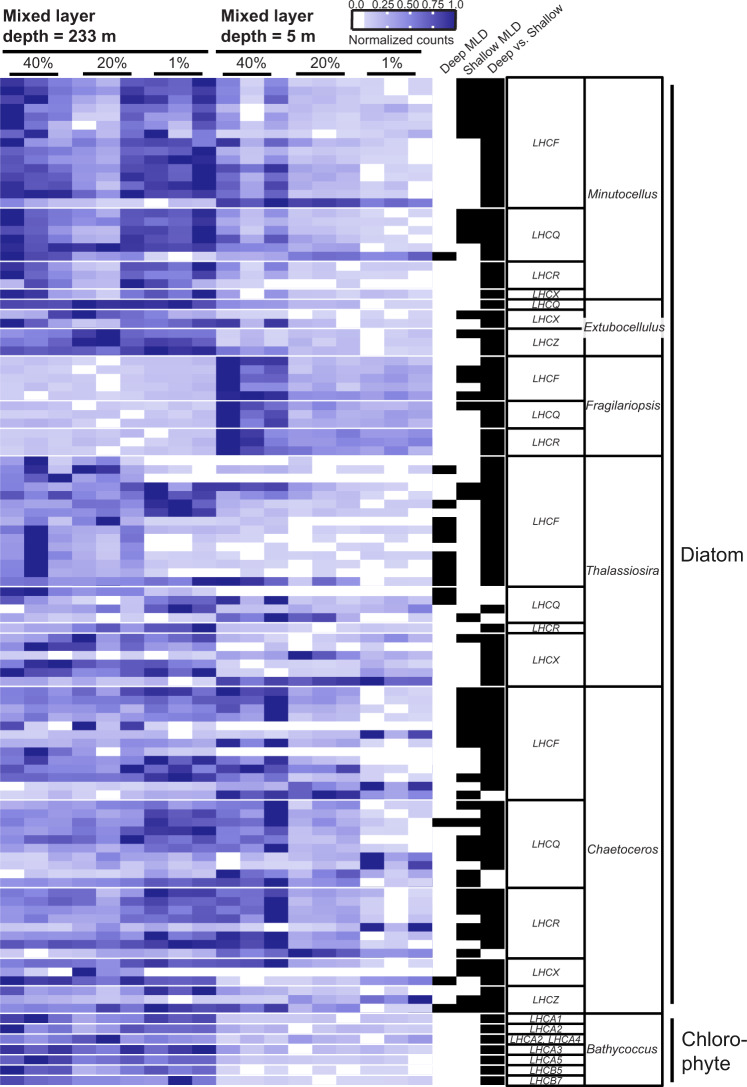
Phytoplankton genera have distinct LHC transcriptomic patterns in response to stratification. Heatmap of normalized read counts for chlorophyll-protein complexes (LHCs) normalized by row to reflect relative expression of each gene (see scale bar). Each row represents a unique transcript and each heatmap column represents a triplicate samples from within the euphotic zone at different mixed layer depths (233 m for 24 May and 5 m for 26 May, Fig. 1A). The next three columns denote whether that transcript was differentially expressed (black bar) within the water column from the deeply mixed (“Deep MLD”), shallow mixed layer (“Shallow MLD”), or between water columns from the same light level (40%, 20%, or 1% relative surface irradiance, “Stratified”). Putative light harvesting complex gene names are listed in the next column to the right with genus names indicated in the last column on the right. Broad taxa labels (Diatoms, Chlorophyte) are provided on the far right.

Since shallower MLDs are associated with higher integrated light exposure over the day, we investigated expression of LHCs and their ratio to the core photosynthesis proteins. Diatoms and chlorophytes differentially expressed a suite of LHCs in response to rapid stratification (Fig. 2). We identified 340 unique diatom LHC transcripts, also called fucoxanthin–chlorophyll binding proteins, or FCPs [50] (Supplementary Fig. 3), with the highest amount of unique LHC transcripts within the *Thalassiosira* genus (Supplementary Fig. 4). Based on recent studies, we expected a variety of both up- and downregulation of LHCs [16, 17, 51], whose roles are still being deciphered [50–53]. Three LHC transcriptional patterns emerged when comparing samples collected from deep to shallow MLDs. The first pattern was shared between *Minutocellus*, *Extobocellulus*, and *Chaetoceros* genera, which highly expressed a suite of LHC genes both in deep and shallow MLs (Fig. 2). A second pattern was shared between *Thalassiosira* and *Bathycoccus*, which highly expressed their assortment of LHC genes within the deep ML but downregulated them within the shallow ML (Fig. 2). The convergence of transcriptional patterns between these two genera from different phyla was not correlated with amino acid similarity of light harvesting complexes (Supplementary Fig. 5). The third pattern was only employed by *Fragilariopsis*, which expressed its suite of LHC genes at the highest level at 5 m depth within the shallow ML (Fig. 2).

The variety of LHC transcriptional strategies to rapid ML shallowing likely reflects multifunctional LHC proteins within a species and/or different species responses within each genus to enable proliferation in environments with dynamic light availability. Several studies have described a variety of LHCX and LHCF proteins in some cultured diatom species to be both upregulated and downregulated in response to high light exposure [54], reflective of distinct roles and responses. A subset of LHCX proteins increase their ability to transfer excitons to quenching pigments when luminal pH is lowered [55], which may trigger unique gene expression feedback within each genus. Different *Thalassiosira* species have been shown to increase accessory pigment to chlorophyll ratios within hours of shifting from low to high light [56]. Since LHC proteins bind to these accessory pigments, it is logical to infer LHC gene expression could also change within hours of high light exposure. The aforementioned possibility of lateral displacement of water masses may have contributed to the observed intra-genus LHC transcription variability by introducing a heterogenous community.

**Fig. 3:**
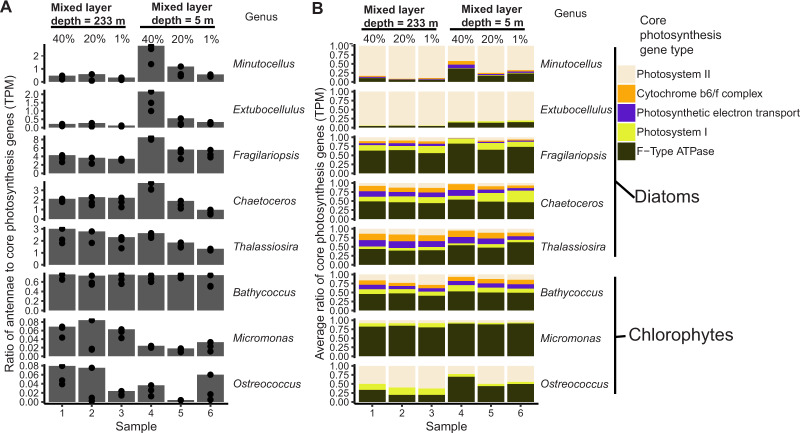
Mixed layer shallowing induces different investments in the relative ratios of photosystem LHC to core photosynthesis genes. **A**. Bar height represents the highest triplicate ratios of LHC: core photosynthesis transcripts for different taxa (genera on far right). Points represent TPM ratios from each triplicate sample. Each column location in **A** and **B** represent samples corresponding to relative surface irradiance (40%, 20%, 1% surface irradiance) within different mixed layer depths (from Fig. 1A and indicated on top axis). **B**. Bar height represents the relative transcript abundance of each core photosystem gene class (indicated by different colors) for different taxa (genera on far right), as determined from the mean of triplicate TPM’s. Note that the relative contribution of PSII transcripts is generally lowest in the SLM.

Following electron flow from LHCs to core photosynthesis proteins, we next compared transcriptional patterns in the ratio of LHCs to core photosynthesis genes (photosystem I and II subunits, Cytochrome b6/f complex, photosynthetic electron transport and F-Type ATPases), which revealed photoacclimation strategies in response to ML shallowing. Two patterns emerged. In four of the five diatom genera analyzed (*Minutocellus, Extobocellulus, Fragilariopsis, Chaetoceros)*, the ratio of LHC:core photosynthetic transcripts increased in the shallow ML (Fig. 3A). *Thalassiosira* sp. and chlorophyte taxa, on the other hand, did not alter their LHC:core photosynthesis transcript ratio in response to ML stratification (Fig. 3A). The inter-genus differences of LHC transcription may reflect different functionalities of LHC proteins (i.e., photon harvesting or photoprotection) or different light harvesting strategies between genera. For example, *Bathycoccus* downregulated all of its LHCs within and below the shallow ML, while *Chaetoceros*’s LHC expression was higher within the shallow ML, gradually downregulating as light decreased in the shallow ML euphotic zone (Fig. 2). This could reflect a slower, more constitutive and steady accumulation strategy of *Bathycoccus* or other green algae than diatoms, which typically doubles less than once per day in culture [57]. In contrast, the more rapid and dynamic strategy employed by *Chaetoceros* can support much higher growth rates in the presence of high light, allowing cells to potentially divide more than twice per day [58]. Additional time-resolved studies are needed to reveal if the differences observed in predawn LHC transcription converge or further diverge between genera as light intensities increase through the day.

### Common LHC and core photosynthesis transcription strategies

Although there was high inter-genus variability in LHC transcriptional strategies, the ratio of transcripts for total LHC to core photosynthesis, as well as LHC subtypes, showed more unified trends across genera. We also noted that the relative ratio of PSII transcripts to other core photosynthesis transcripts decreased across almost all genera in response to rapid stratification (Fig. 3B). In addition, the ratio of transcripts encoding for different LHC subtypes was largely the same within both MLDs (Supplementary Fig. 6).

**Fig. 4:**
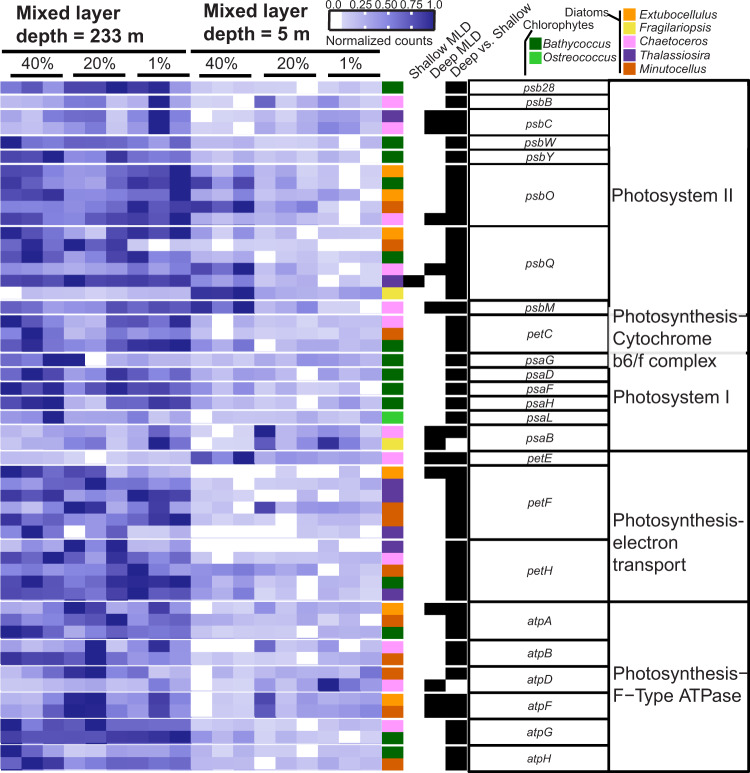
Core photosynthesis genes are downregulated in response to stratification across genera. Heatmap of normalized read counts normalized by row to reflect relative expression of each gene (see scale bar). Each row represents a unique transcript (colored by genus; see key) and each heatmap column represents a triplicate from within the euphotic zone in different mixed layer depths (233 m for 24 May and 5 m for 26 May, Fig. 1A). The next three columns denote whether that transcript was differentially expressed (black bar) within the water column for deeply mixed (“Deep MLD”) or shallow mixed layers (“Shallow MLD”), or between water columns from the same light level (40%, 20%, or 1% relative surface irradiance, “Deep vs. Shallow”). Putative protein names are listed in the next column to the right, with gene module or pathway provided in the last column.

Patterns in core photosystem genes generally supported our hypothesis as expression was downregulated upon stratification across multiple phytoplankton genera, with a few exceptions. A few genes from PSII (*psbO*, *psbQ*) were relatively highly transcribed in the 40% irradiance depth of the recently shallowed ML in diatoms (Fig. 4). Only one core photosynthesis gene (*psbQ* in *Thalassiosira*) was differentially expressed within the deep ML (Fig. 4). The downregulation of core photosystem genes and the lowered ratio of PSII genes to other core photosystem genes suggest a photoacclimation strategy aimed at limiting the flow of electrons through the core photosystem in a higher light environment. The downregulation of core photosystem genes from populations within the stratified ML of this study is corroborated by phytoplankton cultures shifted from low to high light in the first 24 h [14, 59]. This agrees with the MLD shallowing by about 15:00 h the prior day (May 25th), exposing communities to higher daily irradiance until approximately 22:00 h, when PAR decreased below 10 µmol photons m^2^ s^−1^ (Fig. 1A, B). In stably stratified environments, a variety of phytoplankton taxa downregulate core photosystem genes by mid-day [25–29]. This strategy is employed in part because excess photons and rapid oxygen generation can cause oxidative damage [60, 61]. Accordingly, we extended our analysis to evaluate changes in genes encoding proteins that can mitigate these oxidizing effects through non-photochemical quenching (NPQ) [13] and oxidant-scavenging.

### Muted carotenoid biosynthesis and oxidant-scavenging enzyme transcriptional response

**Fig. 5:**
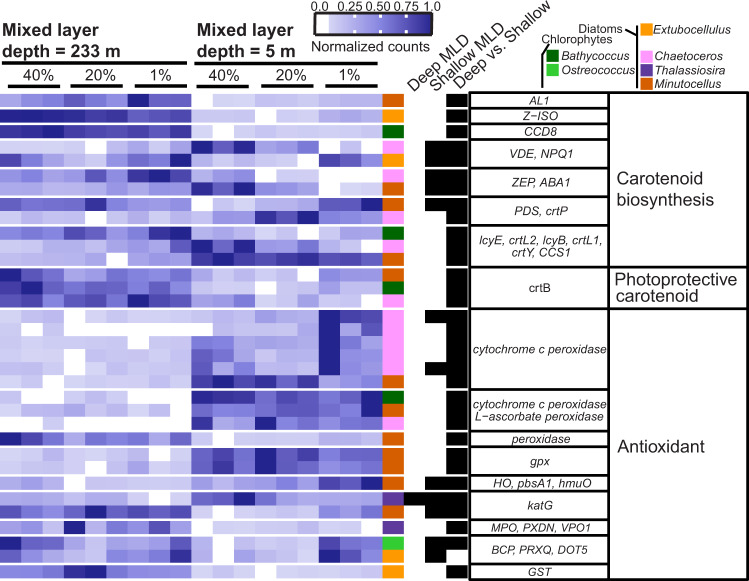
Divergent carotenoid and antioxidant transcriptional response to stratification across genera. Heatmap of normalized read counts normalized by row to reflect relative expression of each gene (see scale bar). Each row represents a unique transcript (colored by genus; see key) and each heatmap column is a triplicate from within the euphotic zone in different mixed layer depths (233 m for 24 May and 5 m for 26 May, Fig. 1A). The next three columns denote whether that transcript was differentially expressed (black bar) within the water column for the deeply mixed (“Deep MLD”) or shallow mixed layers (“Shallow MLD”), or between water columns from the same light level (40%, 20%, or 1% relative surface irradiance, “Stratified”). Putative protein names are listed in the next column to the right, with gene module or pathway denoted in the last column.

Based on the bulk community changes in pigment ratios (Supplementary Fig. 2I), we expected a large number of genes encoding for protective LHCX proteins, carotenoids, and antioxidant enzymes to show significant increases in expression. LHCX proteins, and the carotenoids contained within them, have been shown to quench excess exciton energy during high light exposure [16]. Contrary to our hypothesis, a large proportion of carotenoid biosynthesis and oxidant-scavenging genes detected across the community were not differentially expressed during our predawn sampling of different MLDs (Supplementary Fig. 7). Some oxidant-scavenging and carotenoid biosynthesis genes were more highly expressed in the deep ML, while others were highly expressed in the shallow ML, or even at only a single light intensity (Fig. 5). Carotenoids with only photoprotective functions were an exception, as a small group of these genes were consistently upregulated in several genera (*Minutocellus*, *Chaetoceros*, *Bathycoccus*) in the deeply mixed water column (Fig. 5). This could be indicative of a taxa-specific photoprotection response unique to transient lighting experienced within a deep ML.

The lack of a widespread carotenoid biosynthesis response and only moderate antioxidant responses may have multiple explanations. One explanation is that carotenoid and antioxidant upregulation was muted predawn and would have increased during the day when increased turnover of these molecules is needed. This explanation is supported by the large number of transcripts associated with protective mechanisms that were detected but not differentially expressed in the comparisons used in this study (Supplementary Fig. 7). It is further supported by observations of increased carotenoid transcription after sunrise [62], and increased diatom NPQ [13, 16, 54, 56] and LHCX transcription [63, 64] within one hour of high light exposure. An alternative explanation for a lack of upregulated genes for carotenoid biosynthesis is that the fucoxanthin and diadinoxanthin biosynthesis pathways are not fully mapped [14] and consequently were missed in our analysis.

### Shared central carbon transcriptional strategies

**Fig. 6:**
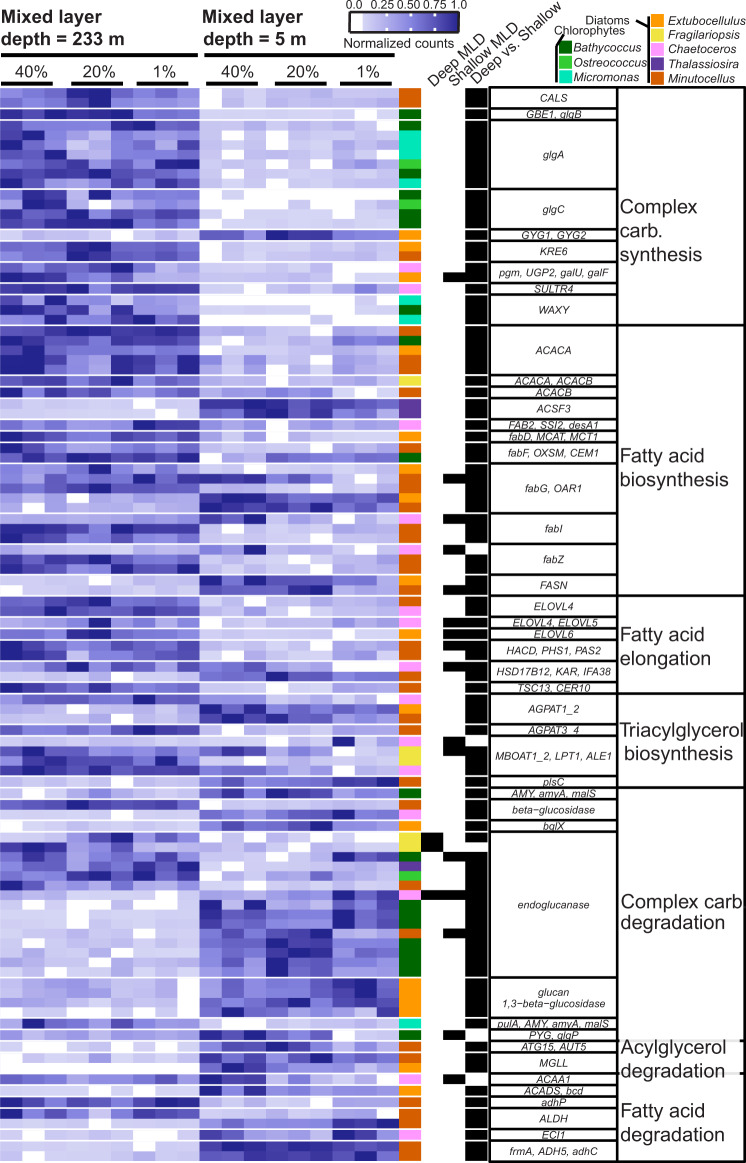
Carbon storage transcription shifts to catabolism in a shallow mixed layer. Heatmap of normalized read counts (DESeq2) from selected unicellular phytoplankton genera (color coded; see key) across all samples, further normalized by row to reflect relative expression of each gene. Each row represents a unique transcript (see scale bar) and each heatmap column is a replicate of 6 samples from within the euphotic zone in different mixed layer depths (233 m for 24 May and 5 m for 26 May). The next three columns denote whether that transcript was differentially expressed (black bar) within the water column for deeply mixed (“Deep MLD”) or shallow mixed layers (“Shallow MLD”), or between water columns from the same light level (40%, 20%, or 1% relative surface irradiance, “Stratified”). Putative protein names are listed in the next column to the right, with gene module or pathway denoted in the last column.

Carbon storage and central carbon transcripts of phytoplankton populations reveal an energy-conserving metabolism in the deep ML compared to a rapidly dividing growth state in the shallow ML. Transcripts involved in anabolic carbon metabolism were generally upregulated in the deep ML. Diatoms generally downregulated their fatty acid synthesis genes and upregulated their fatty acid catabolism genes in the shoaled ML with an exception being transcripts coding for GYG1/GYG2, ACSF3, and fabG/OAR1 (Fig. 6). Chlorophytes uniformly upregulated complex carbohydrate synthesis genes and downregulated carbohydrate degradation genes in the deeply mixed water column (Fig. 6). Diatoms exhibited larger variability in their expression of carbohydrate synthesis and degradation transcripts (Fig. 6). We detected the largest number of unique carbon energy storage genes in the *Bathycoccus* genus **(**Supplementary Fig. 8), suggesting an important role for complex carbohydrate management in relation to MLD. Most phytoplankton taxa shifted their central carbon metabolism towards increased NADPH production via upregulation of the pentose phosphate pathway (PPP) upon ML shallowing (Supplementary Fig. 9). We argue this transcriptional commitment towards catabolism of complex carbohydrates and PPP allows cells to increase their capacity for biosynthesis of nucleic acids and other cell components in support of increased growth rates (Fig. 6 and Supplementary Fig. 9). Conversely, upregulation of carbon storage genes and Calvin cycle genes would allow cells to maximize energy efficiency in the light-limited environment of the deep ML (Fig. 6 and Supplementary Fig. 9).

**Fig. 7:**
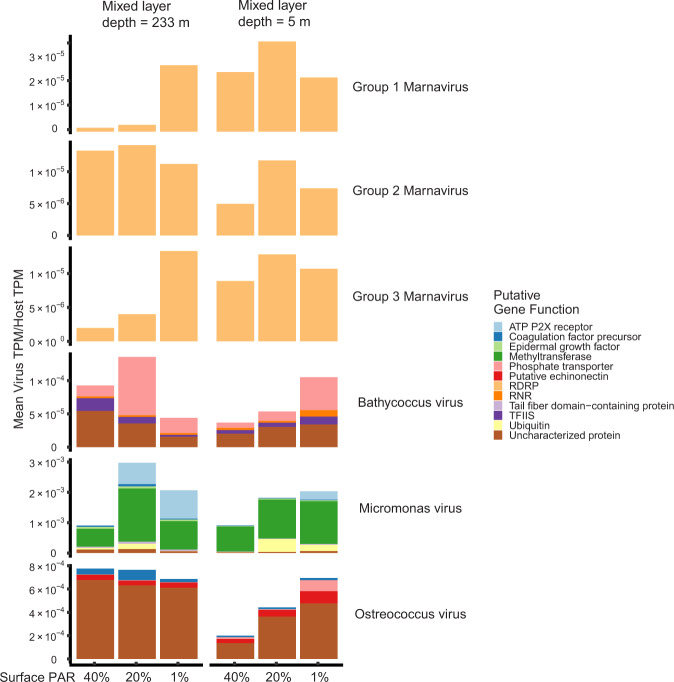
Relative abundance of putative diatom and chlorophyte viruses for deeply mixed and shallow mixed layers. Bar graph height is the average ratio of virus gene TPM to total host TPM (from triplicates) for populations at different relative surface irradiances within the euphotic zone and for different mixed layer depths (see top x-axis label). Note different y-axis scales. Bars are filled with the average ratio of each putative gene class (color coded; see key). Viral genes were filtered based on the closest NCBI matches to other virus genes (Supplementary Table 3), and the translated amino acid sequences and putative functions of these genes (Supplementary Table 4). Putative Marnavirus (diatom-infecting virus) groups were determined via phylogenic tree (Supplementary Fig. 10).

Phytoplankton populations in the shallow ML exhibited a rapid increase in bulk chlorophyll A (Supplementary Fig. 2H) and cell concentration [4], along with a decrease in chlorophyll fluorescence per biomass [10], implying that the increase in light had a stimulating effect on fixing carbon. This is somewhat counterintuitive given that Calvin cycle genes were downregulated in the shallow ML (Supplementary Fig. 9). However, downregulation of Calvin cycle genes may have occurred for several reasons. One explanation is that Calvin cycle gene expression could have been constitutively primed during lower daily light levels in the deep ML to maximize carbon fixation and carbon storage anabolic pathways when briefly mixed to surface waters (Fig. 6) (i.e., “make hay while the sun shines”) [65]. This idea agrees with previous studies [25, 26] in which phytoplankton require a few hours of sunlight to downregulate Calvin cycle genes, and shift central carbon metabolism towards NADPH precursors for biosynthesis of nucleic acids and other cell components required for faster growth. Alternatively, Calvin cycle downregulation may be a virus defense strategy [66, 67] or viral alteration of host metabolism [68–71], which are logical given the increased virus:host ratios observed in some diatom groups within the shallow ML (see below; Fig. 7).

### Viral infection response to mixed layer shallowing

**Fig. 8:**
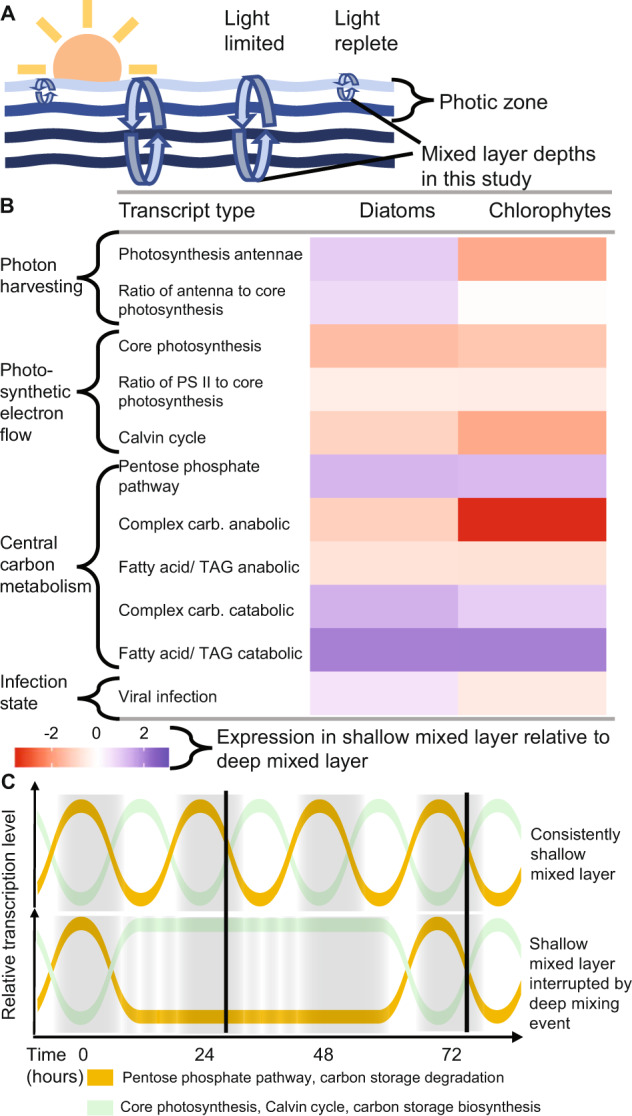
Summary of shared inter-phyla transcriptional responses to changes in MLD and proposed diel model. **A** Conceptual illustration of transient mixing encountered in this study. Light blue denotes the depth of the euphotic zone while dark blue denotes depths where photosynthetically active radiation is below 1% of the surface value. Arrow size indicates the depth of mixed layers analyzed in this study. Note that respective deep and shallow ML conditions are associated with light-limited or light-replete conditions. **B** Phylum-level (diatoms, chlorophytes) analysis of gene expression for pathways (rows) associated with photon harvesting, photosynthesis, central carbon metabolism, and virus infection (see methods for details). Boxes are filled with the log_2_ fold change between samples within the shallow mixed layer (40% relative surface light) relative to samples within the deep ML (40%, 20%, and 1% relative surface light). Colors are shaded by median TPM ratio (“Viral Infection”) or median value of differentially expressed transcripts (remaining categories). “Photosynthetic electron flow” and “Central carbon metabolism” have the same overall direction comprising the shared inter-phyla transcriptional response, which informs the categories used in (**C**). **C** Proposed conceptual model illustrating how a deepening ML would interrupt oscillating diel transcription patterns of core gene classes (see color key), based on expression data from previous field studies using similar markers as in our study [25, 26, 31]. Upper panel represents observed transcription patterns within a stable, shallow mixed layer, whereby the relative levels of expression of core gene sets oscillate several hours after sunlight exposure or darkness [25, 26, 30, 31]. The bottom panel illustrates how the lower integrated sunlight exposure associated with deep MLs that exceed the euphotic zone leaves gene sets in respective high and low transcriptional levels (as observed in this study; for illustration purposes the deep mixing event is assumed to occur at 0 h). We posit that the expression patterns are being driven by cell division and resources from the previous day’s production, rather than a true circadian-driven expression, which would continue even during deep mixing but with a diminution of values with each oscillation. Gray boxes represent time without sunlight due to diel cycle or deep mixing. Note that lower integrated light exposure (represented by thin white lines) associated with a deep ML necessitates greater economy of photosynthesis and carbon fixation. Vertical axis scales represent relative expression levels under each condition (shallow versus disturbed ML) and may not be the same between the two rows. Black lines indicate predawn sampling times employed in this study. Groups of genes are colored (green or orange) by their respective positive or negative transcriptional response to ML shallowing and higher integrated sunlight exposure.

Given that our previous work reported decreases in bulk virus:cell ratios upon ML shallowing [4], we probed the community to see if the ML-induced, central metabolic changes were reflected in transcript markers of active viral infection across genera. On a phylum-wide perspective, diatoms generally showed transcriptomic evidence of more infection upon ML shallowing, whereas chlorophytes showed lower molecular signatures of infection (Figs. 7, 8B). Our methodological rationale, based on prior studies [72, 73], was that the degree of infection can be inferred from the relative ratio of intracellular virus transcripts to host transcripts. We note that our samples consisted of biomass collected onto 0.8 µm pore-size filters and our reads were enriched from polyadenylated transcripts, so detected virus transcripts would be primarily associated with expression (and replication) within cells. It is possible (but less likely) that a portion of virus reads may have come from free viral particles outside of cells collected onto 0.8 µm pore-size filters with cells during sampling.

The viral infection response to stratification varied by taxa, reflecting a partitioned succession of virus infection. Diatom cells appeared to be under greater viral pressure than chlorophytes upon rapid ML shallowing, which may further increase with prolonged stratification [4]. Since the most abundant diatom genera present in our samples (Fig. 1D) have no isolated and sequenced viruses in culture, viral taxonomic identity was inferred by sequence similarity to previously cultured and sequenced diatom viral genomes which fall within the *Marnavirus* genus [74] (Supplementary Fig. 10). Given most of the putative virus sequence identified in this study appeared more closely related to non-phytoplankton virus groups whose hosts’ transcripts were also detected (Supplementary Fig. 10, “Other”), it is possible our conservative analysis failed to identify some novel diatom-infecting viruses. It is also possible that these viral transcripts originate from viruses infecting other organisms (e.g., seaweed, arthropods, or other animals), for which host transcripts were also detected (Fig. 1C, “Other”). In the surface water, two out of the three diatom virus groups (*Marnavirus* Groups 1 and 3) increased in relative transcript abundance as the ML shallowed, while one decreased in response to the ML shallowing. (Fig. 7). Also in the surface water, the ratio of virus to host trasncripts within *Bathycoccus* and *Ostreococcus* genera decreased, as the ML shallowed, while *Micromonas* was unchanged. All chlorophyte viruses had lowered virus:host transcript ratios in the shallow ML (Fig. 7), implying that virus replication within this group was not stimulated under these conditions. The decrease in chlorophyte virus:host ratios supports our hypothesis and is consistent with observed decreases in the concentrations of 100–200 nm diameter, dsDNA-containing viruses in the same surface water [4] (this excludes the detection of ssRNA and ssDNA containing diatom viruses less than 50 nm) [75, 76]. Despite temporal lags between the presence/abundance of cell-associated viral transcripts, host lysis, and viral particle production, our data show that viruses were not actively replicating in chlorophyte taxa between the deep and shallow MLDs (Fig. 7B**)**. Using the closest protein homology of translated transcripts (Supplementary Tables 3 and 4), we did not detect major capsid protein expressed above the threshold used in this study. From this observation we hypothesize that chlorophytes in the deep MLD were either in an early stage of lytic infection, or in a lysogenic/ pseudo lysogenic state. We speculate that some of the expressed viral genes were enhancing host nutrient acquisition and growth under prevailing conditions. For example, a putative viral phosphate transporter gene was expressed higher in the deep ML than the top of the shallow ML (Fig. 7). Similar beneficial functions may occur in other systems, such as the phosphate permease transporters found in *Emiliania huxleyi* virus genomes [77] or cyanophage photosystem protein expression in cyanobacteria hosts [78]. This mechanism could be further evaluated in future fieldwork using single cell sequencing and molecular assessments of extracellular virus production at higher temporal resolution and over extended time series as water mass MLs shallow and deepen.

Our analysis suggests that different host and virus successions likely occur on short time scales during transient late-spring mixing and shallowing events in the North Atlantic and implicates the role of viral pathogens in the classic bloom and bust successional sequence of phytoplankton communities in regions of the North Atlantic [5, 7, 79]. Diatoms and other eukaryotic phytoplankton have been shown to exhibit lysis during nutrient deprivation or some other physiological trigger than occurs at the end of a growth curve [74, 80, 81], which may be a sign of a temperate viral life cycle. Indeed, recent work has shown that physiology-dependent temperate dynamics operate in coccolithophores (previously thought to be strictly lytic) [80] and lysogeny has been hypothesized to operate in other eukaryotic algae (including diatoms and chlorophytes) given the constraints on physical encounters and viral decay [80]. We previously showed that total community viral accumulation (all dsDNA viruses >0.05 and <0.2 µm in diameter within the euphotic zone) is highest during periods of the North Atlantic bloom with nutrient limitation and reactive oxygen stress biomarkers [4]. In this study, macronutrients were not limiting (Supplementary Fig. 2E–G) but light was limiting in some samples, and reactive oxygen stress was likely high based on our previous bulk community analysis [4]; together they suggest a combination of saturated light and reactive oxygen stress may trigger viral lysis in diatom communities. Chlorophyte viruses apparently had an opposite trigger; low light possibly triggered early stages of lysis or at least increased viral transcript abundance (Fig. 7). To date, it is unknown what specific environmental stresses can trigger viral replication or induction but our results highlight important areas of investigation with implications to viral ecology. The impact of rapid changes in MLD and other physical drivers on subcellular molecular interactions are a ripe area for investigation in viral ecology and bloom progression, as predation pressure is likely a key force shaping the annual bloom in the North Atlantic [1, 4].

### Phylum-level responses to oscillations in mixed layer depth

We synthesized a phylum-level summary (Fig. 8B) of the transcriptomic responses to ML stratification to provide ecophysiological context for our findings. Diatoms increased their ratio of LHC:core photosynthesis transcripts when trapped in the shallow ML, while chlorophytes decreased this ratio. Anabolic pathways used for carbon storage (e.g., biosynthesis of polysaccharides and fatty acids) and Calvin cycle genes were downregulated across phyla in the shallow ML, instead committing more towards cellular pathways involved in the catabolism of stored resources to support rapid growth. Lastly, virus replication within cells increased overall in diatoms and decreased in chlorophytes, hinting at a differential fate for these phyla if the ML had remained stratified for several more days or weeks [4]. This phylum-level summary of our dataset revealed that diatoms and chlorophytes exposed to high light during the day appeared to retain imprinted transcriptomic patterns when sampled at predawn the following day (Fig. 1B, E). For example, phytoplankton simultaneously upregulated carbon catabolism genes (Fig. 6), downregulated core photosystem genes (Fig. 4), and lowered the ratio of PSII transcripts relative to other core photosynthesis transcripts (Fig. 3), all before sunrise (Fig. 1B). This led us to consider if the previous day’s integrated daily irradiance and associated cell division and carbon production affected the transcriptional state of predawn phytoplankton cells.

Previous studies have shown that phytoplankton within shallow stable MLs begin to upregulate core photosystem transcripts during the night and reach their highest expression at mid-day [30], as lipid stores begin to increase [25] (Fig. 8C). We posit that these observed predawn expression patterns are tuned to the growth and division rates established by light availability from the previous day. In this scenario, cells receive sufficient integrated light exposure and sufficient photosynthetic output, which in turn upregulate the PPP and catabolic carbon pathways to increase cellular NADPH and ATP production adequate to support higher division rates. We argue that a deep mixing event (down to >200 m MLD), like that encountered on the first day of our study, resulted in phytoplankton communities receiving insufficient daily irradiance to support similarly high rates of cell division. Consequently, cells maintained relatively high predawn levels of transcripts related to core photosynthesis, carbon fixation (Calvin cycle) and anabolic energy storage genes and did not increase PPP or catabolic carbon storage genes (Fig. 8C). This strategy allows deeply mixed, light-starved communities to maximize photosynthesis and carbon fixation and conserve fixed carbon over shorter integrated periods of light exposure in deep MLs. In this scenario, energy-starved populations must maintain higher relative levels of mRNA for core photosynthesis, Calvin cycle, LHCs and other metabolic pathways. Although mRNA levels for these key genes remains relatively high during this light limited/ energy limited condition, it’s possible that the higher energy and macromolecular cost of maintaining protein pools during light-limited conditions may be mitigated through decreasing protein turnover and translation rates [82, 83]. Future studies employing a sampling regime with higher time resolution can rigorously test this ‘deep disruption’ conceptual model and better evaluate its ecophysiological interpretation.

## Conclusion

Our metatranscriptomic-based study provides unique insight into the in situ, differential expression of specific genes related to photosynthesis, virus infection and carbon cycling in response to changes in MLDs. Transitioning from a deep to shallow ML state induced a conserved downregulation of core photosynthesis and carbon fixation genes and concomitant upregulation of carbon catabolism genes, along with divergent responses in terms of photosynthetic LHC transcription and viral infection. Biosynthesis of carotenoids that serve as quenching molecules for excess absorbed light energy was only modestly upregulated in the shallow ML, a somewhat counterintuitive observation that may simply reflect predawn sampling times. Comparing our observed phytoplankton transcriptomic responses to previously documented diel expression in stable MLs, we hypothesize that lower daily sunlight exposure during deep mixing events decreases daily carbon fixation and consequently necessitates sustained high levels of transcripts related to photosynthesis and carbon storage. This type of diel response argues that increased light absorption from ML shallowing may signal for increased transcription related to carbon catabolism to support enhanced cell division, a conceptual hypothesis that can be tested in subsequent work. Taken together, our study documents a range of transcriptional strategies related to photon flux and carbon flow across diatom and chlorophyte taxa, reflecting both shared and divergent responses to transient physical mixing events and subsequent stratification.

## Materials and methods

### Physical parameters

All samples were collected as part of the North Atlantic Aerosol and Marine Ecosystem Study (NAAMES) during the May 2016 campaign aboard the R/V Atlantis (AT34), which targeted the “Climax” phase of the annual biomass cycle [3, 4]. Salinity and temperature were collected both from the ship’s CTD and from profiling floats at depths and time intervals (Supplementary Table 2). MLDs were determined as the depth below 5 m at which the Brunt–Väisālä buoyancy frequency (N^2^) was greater than its standard deviation, as calculated previously in other NAAMES studies [8, 10].

### Macronutrients

Inorganic nutrient concentrations (NO_3,_ SIO_4_, PO_4_) were determined previously for this station [3]. Samples from each depth and time interval were gravity filtered directly from the Niskin bottles through inline 47 mm PC filtration cartridges loaded with 0.8 μm polycarbonate filters into sterile 50 mL conical centrifuge tubes and stored at −20 °C for later analysis using the Lachat QuickChem QC8500 automated ion analyzer at the University of Rhode Island Graduate School of Oceanography Marine Science Research Facility (GSO-MSRF).

### Surface irradiance

Satellite-derived daily integrated incident surface irradiance at the study site was derived from MODIS-Aqua satellite broadband PAR products (400–700 nm) from sunrise to sunset for each day within the time period surrounding station occupation. Satellite values were averaged over a 2 × 2 pixel region (1 pixel = 250 m resolution) centered on NAAMES II, Station 4. Comparable, shipboard estimates of daily integrated PAR were calculated from continuously measured irradiance using a Licor (Lincoln, Nebraska) Model LI-189 cosine collector positioned to avoid shading from ship structures. Sampling depths were determined from relative surface PAR (40%, 20%, 1%) from near noon optical profiles of the water column with a Biospherical C-OPS radiometer (San Diego, CA).

### Community pigment measurements

Whole seawater samples from each depth and time interval (Supplementary Table 2) were collected in CTD Niskin bottles and vacuum filtered onto 25 mm GF/F filters (Whatman Little Chalfont, Buckinghamshire, UK). Filters were immediately stored in liquid nitrogen after filtration and were kept in liquid nitrogen or at −80 °C until sample analysis. High performance liquid chromatography was performed at the NASA Goddard Space Flight Center, following predetermined quality assurance and quality control protocols [84, 85].

### RNA processing, sequencing and assembly

Whole seawater samples from Niskin bottles were collected via vacuum filtration onto 47 mm, 0.8 µm pore-size polycarbonate membrane filters (ATTP, Millipore) [4]. Filters were flash frozen in liquid nitrogen at sea at the time of sampling and were stored at −80C until processed. Filters were cut in half on dry ice with a sterile scalpel. with one half used for RNA extraction in this study and the other half used for prior caspase and metacaspase enzymatic activity assays [4]. RNA was extracted with Trizol (Invitrogen, Waltham, MA) without modifications. PolyA-enriched messenger RNA was reverse transcribed using Invitrogen SuperScript II Reverse Transcriptase at the Rutgers Genome Cooperative. Libraries were prepared from this cDNA (TruSeq RNA Library Prep Kit v2. Illumina, San Diego, CA) and sequenced using a HiSeq system (Illumina) at 2 × 150 base pairs at Genscript in Edison, NJ.

The raw reads from all samples were searched for sequencing adapters and low-quality nucleotides which were trimmed using CLC Genomic Workbench (version 20.0.4, Length Fraction = 0.8, Similarity Fraction = 0.9, only uniquely mapped reads) [86]. The trimmed reads were assembled using Trinity (version 2.12.0) [87] yielding 11,046,104 contigs. Protein coding regions within the metatranscriptome assembly were predicted with TransDecoder [88]. To reduce redundancy in the metatranscriptome, the predicted coding regions were clustered at 95% identity using CD-HIT [89] which resulted in 2,053,751 sequences. Trimmed reads from each sample were then mapped to the assembly with CLC Workbench and only uniquely mapped reads were counted. Contigs with less than 50 total mapped reads across all samples were filtered out resulting in 673,142 contigs that were used for downstream analysis. Functional annotations were identified using EggNOG [90].

### Relative abundance of taxonomic groups

To assign taxonomic identification to each contig, a custom local protein database was used, incorporating: the re-annotated [91] MMETSP [92]; genes from Tara Ocean contigs with taxonomic identifiers identified by MetaEuk [93]; MaTOU [45]; and Uniref100 (updated May 2020) databases. Proteins were aligned with DIAMOND (v. 2.0.11, ultra-sensitive mode) [94]. A bit-score cutoff of 50 was used. The phylogeny assigned to the top bit-score of each alignment in this custom database was kept for further analysis. One hit for each phylum was kept if a protein sequence mapped to multiple algal phyla, given contigs could have been assembled from multiple phylogenetic phyla.

The top twenty representative genera were determined based on summed transcripts per million (TPM) per sample. TPM was calculated by dividing the read counts of each transcript by the putative gene length in kilobases (reads per kilobase). Every read per kilobase was summed for each sample and divided by 1,000,000 to obtain a scaling factor. Reads per kilobase were divided by each sample’s scaling factor to obtain TPM corresponding to each read and sample.

### Differential expression analysis

Transcripts were grouped at the genus level before passing raw counts to DESeq2 [95] (version 1.28.1) in order to correct for the effect of one taxonomic group increasing in biomass or expressing higher amounts of mRNA per cell between sampling dates; this is has been shown to skew the amount of up and downregulated genes [96]. DESeq2 was used to determine if a gene was significantly changed (False discovery rate/FDR adjusted *p* value <0.001 and log_2_ fold change > |1|). Three groups of comparisons were used to search for significant changes in gene expression within the deeply mixed layer; within the shallow mixed layer; and between respective depths for deep and shallow mixed layers (Fig. 1F). Normalized read values from DESeq2 were used to make heatmaps from subsets of significantly altered genes (Figs. 2, 4, 5, 6 and 8).

### Selection of genes involved in physiological processes

Predicted KEGG terms generated by EGGNOG were used to annotate functional genes by genus. Photosynthesis (ko00195), carotenoid biosynthesis (ko00906) and photosynthesis LHC proteins (ko00196) reference pathways were used, along with a custom set of oxidant-scavenging proteins obtained from other research articles [14, 74]. Only significantly changed genes (adjusted *p* value of <0.001 and log_2_ fold change > |1|) in at least one of the three categories of comparisons were used for expression analysis of key gene pathways related to MLD (Figs. 2, 4, 5, 6 and Supplementary Fig. 9), while all genes were included in analysis of the proportion of key genes significantly changed (Supplementary Figs. 4,  7, and 8).

KEGG terms for light harvesting complex proteins in diatoms do not reflect the functional diversity (i.e., LHCA1 is annotated for many diatom proteins) but their diversity is better reflected in a group of closely related group of proteins called fucoxanthin–chlorophyll protein (FCPs). To discern diatom FCPs in our dataset, each class of FCP proteins found in literature according to Kumazawa et al. [50] (LHCX, LHCF, LHCR, LHCQ, LHCZ) was first downloaded from UniProt. A HMMprofile (HMMer version 3.3) was constructed from each protein class, followed by a HMMsearch to find matches within our diatom contigs. Bitscores under 50 were removed. IQTree (version 1.6.12, ModelFinder Plus, 1000 bootstrap replicates and bnni option, model VT + R8 chosen according to BIC) was used to align these sequences along with the previously annotated proteins. The corresponding groups of FCP proteins were determined based on their respective group phylogeny in the tree (Supplementary Fig. 3).

To determine catabolic and anabolic storage molecule reactions, genes annotated as starch and sucrose metabolism (ko00500) KEGG pathway, as well as glycogen biosynthesis (M00854) and degradation (M00855) modules. KEGG reactions were used to determine the anabolic or catabolic nature of each term. Ambiguous directionality of reactions was labeled when appropriate (Supplementary Fig. 9).

### Identification of putative diatom and chlorophyte viruses

Diatom viruses were identified by using HMMER HMMscan with a custom database assembled from RNA-dependent RNA polymerase similar to Kranzler et al. [74]. A bit-score of 50 was used as a cutoff. Since viruses from cultured representatives form most of the top genera have not been isolated in culture, a phylogenetic tree was constructed using a ClustalW alignment of the RDRP sequences to confirm the relationship of viruses detected in our study to sequenced virus groups. RAxML web server [97] was used to construct this tree and provide bootstrap support values (model = WAG + F + G4, bootstraps = 100). Our reads, which included a variety of contig and gene lengths, were incorporated using GUPPY’s (https://bio.tools/guppy) and pplacer [98] (v 1.1 alpha19) (Supplementary Fig. 10). Nucleocytoplasmic viral contigs (Chlorophyte viruses) were aligned with PfamScan (cutoff *e* value < 0.001) to determine putative function and closest domain or family. Each domain on the same contig with a significant alignment was reported. Contigs with no matches to PfamScan were blasted against NCBI (BLASTp) to infer putative function based on the top hit (Supplementary Table 3).

### Relative virus abundance

Since most diatom groups analyzed in our study do not have a cultured virus represented in databases, the total virus TPMs were divided by the total diatom TPMs per sample (as in Kranzler et al.) [74] to give a broad diatom infection metric. Since cultured representatives of chlorophyte viruses have published genomes, the sum of TPMs from each type of Prasinovirus transcript detected (*Micromonas* virus, *Ostreococcus* virus, *Bathycoccus* virus) was divided it by the TPM of each host genus by sample.

## Supplementary information

Supplementary Information

Supplementary Table 2

Supplementary Table 3

Supplementary Table 4

Supporting Data 1

Supporting Data 2

Supporting Data 3

Supporting Data 4

Supporting Data 5

## Data Availability

Raw sequencing data used in this transcript, along with translated amino acid sequence, TPM for each gene ID and sample, are available at NCBI BioProject PRJNA859122 (GEO Accession GSE208287). HPLC and other metadata from flow through and CTD casts from this station and throughout NAAMES are publicly available at NASA’s SEABASS Earthdata repository https://seabass.gsfc.nasa.gov/search#bio. Supporting Data 1 contains the translated amino acid sequences of all sequences determined to belong to *Minutocellus, Extobocellulus, Fragilariopsis, Chaetoceros, Thalassiosira, Micromonas, Bathycoccus*, and *Ostreococcus* genera. Supporting Data 2 contains the fold change and adjusted *p* value (significantly changed or not) pertaining to the comparisons used in this study and taxonomic annotation to the 8 aforementioned genera. Supporting Data 3 contains the count matrix of all genes detected in this study (including genes not identified as one of the top 8 aforementioned phytoplankton genera), labeled by the sample numbers (Fig. 1A). Supporting Data 4 contains gene IDs of transcripts from the 8 phytoplankton genera analyzed in this study and the closest Kegg term match, if any as determined by EGGNOG. Supporting Data 5 contains the gene IDs, translated amino acid sequence, and LHC type identified in this study (including those not annotated by KEGG, see “Methods”).
