# Supplementary material for: Marine phytoplankton downregulate core photosynthesis and carbon storage genes upon rapid mixed layer shallowing
Source: ISME J. 2023 May 8;17(7):1074–88. doi: 10.1038/s41396-023-01416-x (PMC10284824; doi:10.1038/s41396-023-01416-x)
Supplement: Supplementary file 1 — Supplementary Information [file 41396_2023_1416_MOESM1_ESM.docx]

**[Supplementary Information for “****Marine phytoplankton downregulate core photosynthesis and carbon storage genes upon rapid mixed layer shallowing”**

**Authors**

Ben P. Diaz^1#^, Ehud Zelzion^2^, Kimberly Halsey^3^, Peter Gaube^4^, Michael Behrenfeld^5^, and Kay D. Bidle^1^*

**Affiliations**

^1^ Department of Marine and Coastal Science, Rutgers University, New Brunswick, NJ 08901, USA

^2^ Office of Advanced Research Computing, Rutgers University, Piscataway, NJ 08854, USA

^3^Department of Microbiology, Oregon State University, Corvallis, OR 97331, USA

^4^ Applied Physics Laboratory, University of Washington, Seattle, WA 98105, USA

^5^ Department of Botany and Plant Pathology, Oregon State University, Corvallis, OR 97331, USA3

# Current affiliation: Biotechnology & Bioengineering, Sandia National Laboratories, 7011 East Avenue, Livermore, CA 94550 USA*Corresponding author Kay Bidle; bidle@marine.rutgers.edu

**Supplementary Table 1, Description of Supplementary Tables 3, 4.**

**Supplementary Figures 1-8**

**Supplementary Table 1. Incidence of rapid shallowing and deepening events in the Northwest Atlantic.** Data derives from floating optical profilers throughout the Northwest Atlantic shown in **Supplementary Figure 1**. “Rapid mixed layer deepening” columns denote the number of MLD observations per float (Float ID with total deployment of “Time elapsed days”) within the depth range sampled (see **Supplementary Figure 1A**), where the mixed layer had deepened from above 25 meters (above typical Northwest Atlantic euphotic zone depth of 50m[1, 2]) to below 100 meters or between 50-100 meters (both below typical euphotic zone depth) within 72 hours. “Rapid mixed layer shallowing” columns denote the number of observations where the MLD shallowed to less than 25 meters from either below 100 meters or from between 50 and 100 m within 72 hours. “Metbio003d” was the floating profiler followed in this study. “No rapid MLD shift” column denotes MLD observations that were taken within 72 hours from each other but had not increased by the criteria described above. The column to the right denotes MLD observations where the previous transmittance was more than 72 hours prior, making it impossible to discern if rapid mixed layer shallowing had occurred.

| Float ID | Time elapsed  (days) | Rapid Mixed Layer Deepening (n) | | Rapid Mixed Layer Shallowing (n) | | No rapid MLD shift  (n) | Time points taken >3 days apart (n)  (unable to detect rapid MLD shift) |
| --- | --- | --- | --- | --- | --- | --- | --- |
|  |  | from  <25m to >100m | from  <25m to 50-100m | from  >100m to <25m | from  50-100m to <25m |  |  |
| metbio003d | 581 | 4 | 13 | 2 | 2 | 309 | 81 |
| metbio010d | 246 | 4 | 9 | 4 | 0 | 309 | 28 |
| n0572 | 644 | 0 | 2 | 0 | 1 | 127 | 102 |
| n0573 | 122 | 0 | 0 | 0 | 0 | 86 | 16 |
| n0574 | 479 | 0 | 8 | 6 | 4 | 362 | 164 |
| n0847 | 451 | 0 | 4 | 0 | 1 | 224 | 1 |
| n0848 | 278 | 0 | 1 | 0 | 1 | 164 | 0 |
| n0849 | 443 | 3 | 4 | 1 | 3 | 236 | 1 |
| n0851 | 396 | 1 | 2 | 1 | 1 | 185 | 1 |

**Supplementary Table 3. Closest match of putative Prasinovirus proteins in this study.**

**(Attached as .xlsx)** "Gene" column is the TRINITY ID used throughout this study. "PFAMmatch" column is the closest PFAM family match. If no significant match was found, NCBI BLASTp was used. "Virus Type" column is one of the 3 Prasinovirus types identified in this study. "Description" column is the text description of the closest PFAM or NCBI match. "Accession" column is the best NCBI Accession if no PFAM match was found. "bitscore NCBI" column is the NCBI bitscore to each sequence is no PFAM match was found.

**Supplementary Table 4. Translated amino acid sequence and putative function of virus proteins in this study.**

**(Attached as .xlsx)** "Gene" column is the TRINITY gene ID used throughout this manuscript. "Virus Type" is the type of Prasinovirus or Marnavirus identified in this study. "Description" is the description of the closest functional match.


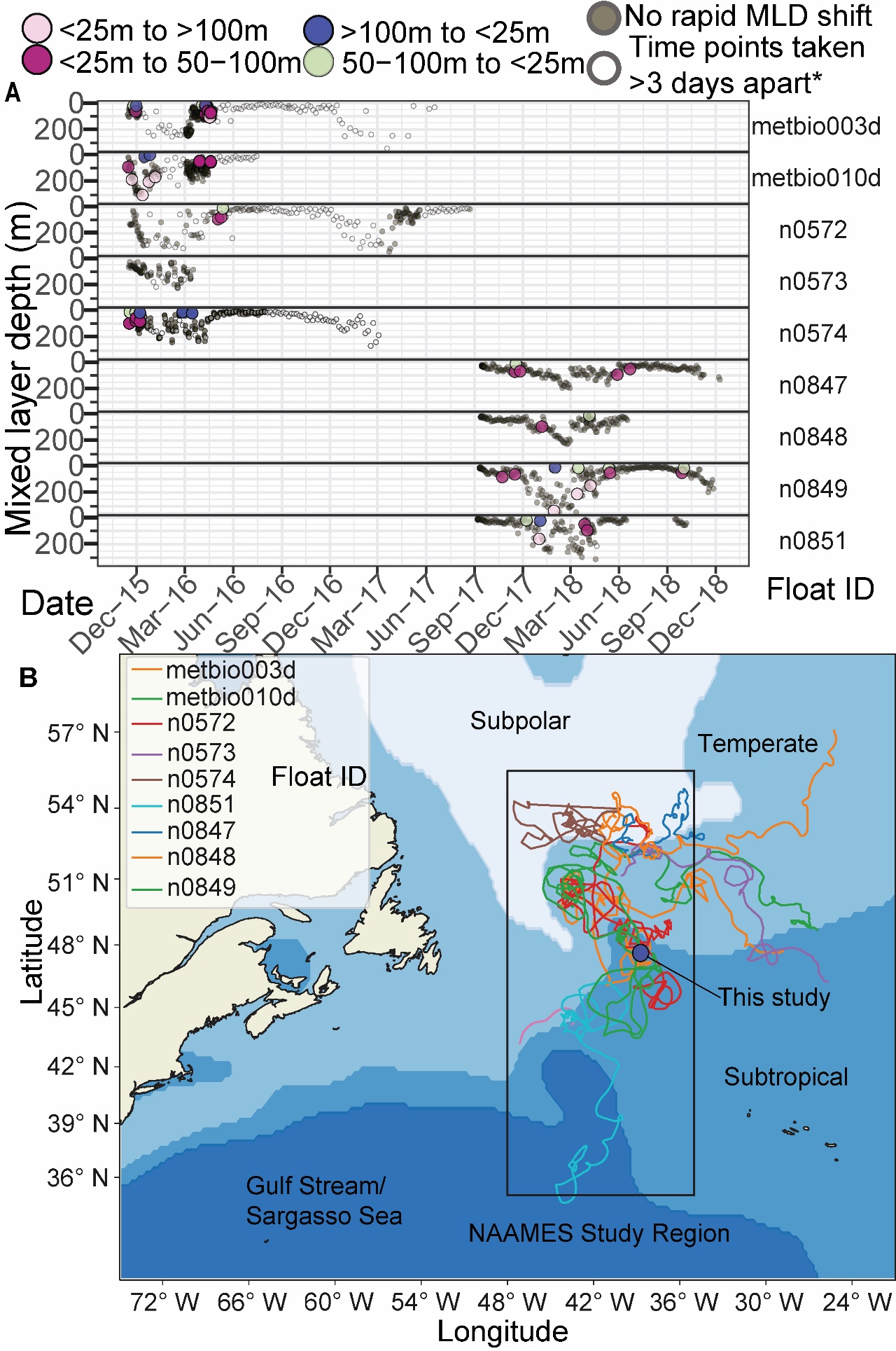


**Supplementary Figure 1. Rapid shallowing and deepening of mixed layer depth occurs throughout the Northwest Atlantic from November to May. A.** Mixed layer depth obtained from floating profilers [3] deployed throughout NAAMES. Points are colored by the extent of their deepening of shallowing. Purple and pink points denote days when mixed layer depth (MLD) depth was under 25m within the previous 72 hours, (within typical N.W. Atlantic euphotic zone, 50 m [1, 2]) and deepened to below 100m (well below typical euphotic zone) or between 50-100m (just below typical euphotic zone). Blue and green points denote a shallow MLD (<25 m) where within the previous 72 hours the MLD was below 100m (blue) or between 50-100m (green). Dark grey smaller points denote no rapid MLD shift as defined above. *Open circles denote sampling intervals larger than 3 d, so rapidity of MLD changes could not be determined. **B.** A regional map of floating profilers corresponding to FloatID’s in **A.** Only samples within the black rectangle were used in this analysis. Water masses colored according to previous analysis [4]. Frequency of rapid mixing events shown in **Supplementary Table 1**. Location of this study is indicated by the blue circle.
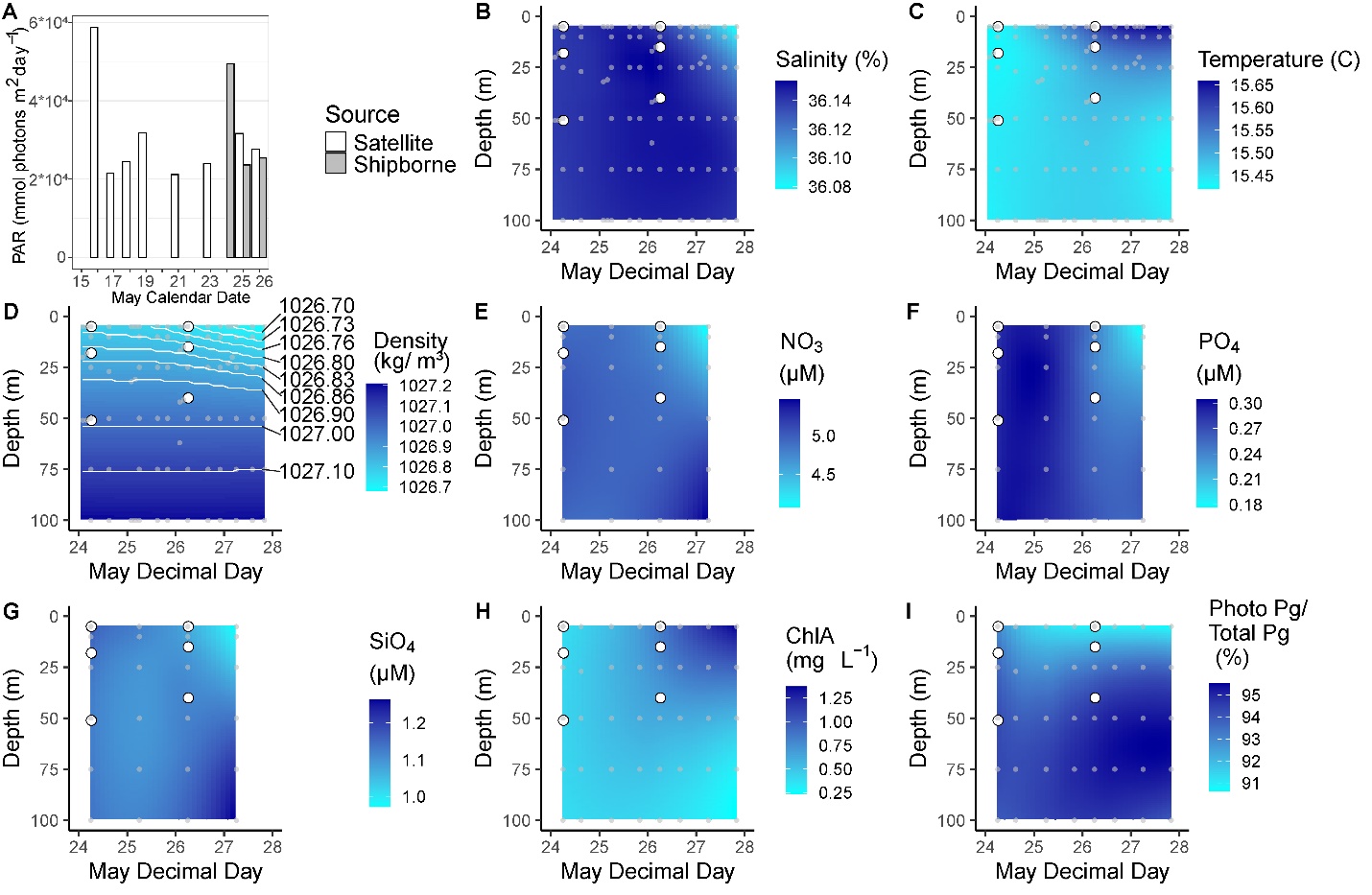
 **Supplementary Figure 2. Irradiance levels, macronutrient concentrations, and bulk pigment properties of samples collected during mixed layer shallowing. A.** In situ and satellite-derived daily irradiance levels (mmol photons m^2^ d^-1^) associated with sampling location (see Methods). Depth profiles of in situ **B** salinity (%), **C** temperature (°C), **D** density (kg/ m^3^) – white contour lines are labeled to show **E** nitrate (µM), **F** phosphate (µM), **G** silicate (µM), **H** Chlorophyll A [Chlide_a + DVChla + Chl a] (mg L^-1^), **I** ratio of photosynthetic pigments to all pigments ([ChlA + ChlB + ChlC + But + Fuco + Hex + Peri; mg L-1] /[ChlA + ChlB + ChlC + But + Fuco + Hex + Peri + Allo + Diad + Diato + Zea + Caro; mg/L-1]; %). Grey dots represent the depth and time of raw datapoints used to generate local polynomial regression fitting in R. X-axis for each plot shows time (May Decimal Day). Open white circles denote sampling depths and times for water used in metatranscriptomic analysis and for which a variety of physiological and biochemical markers have been previously published [5].


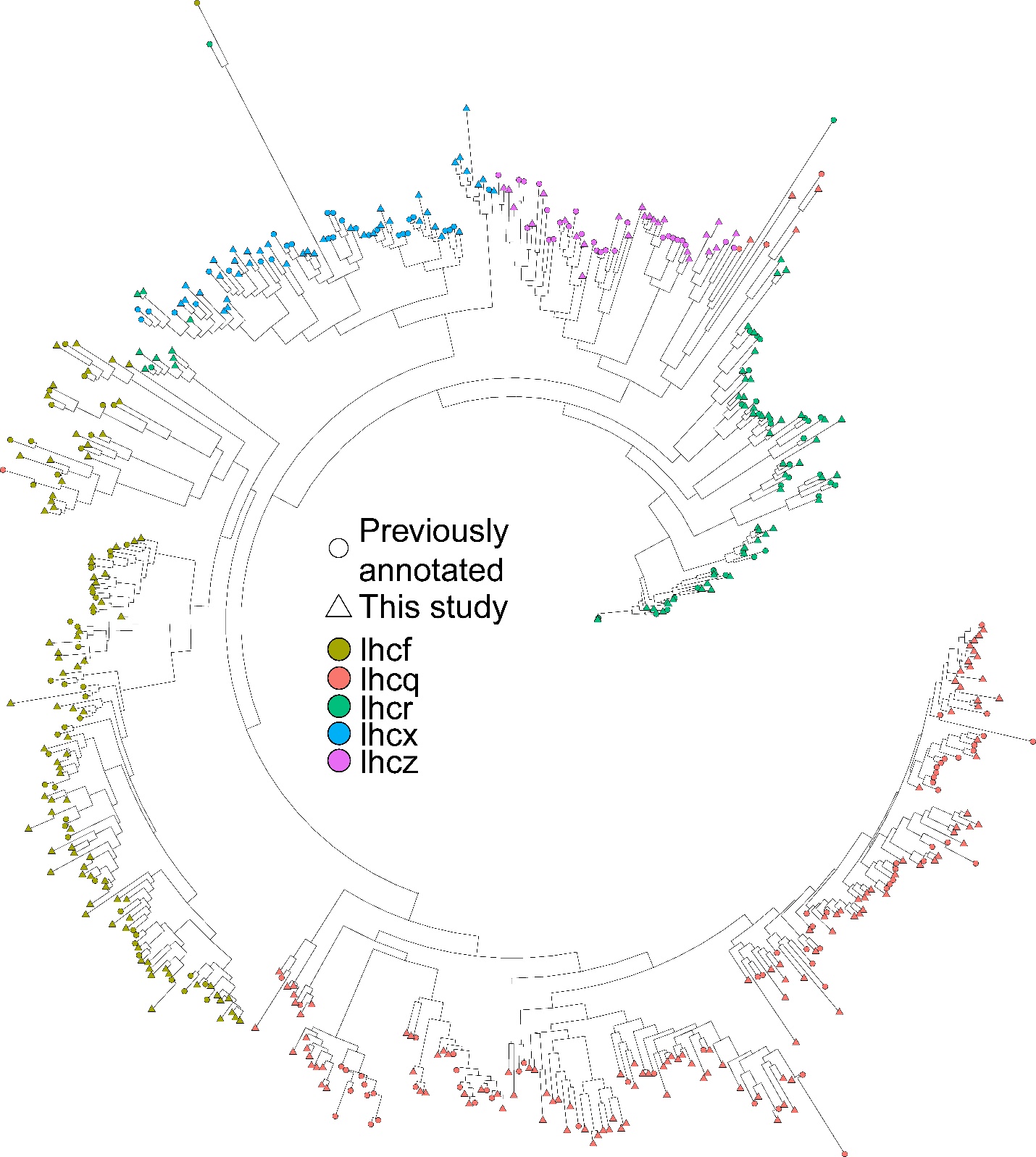
**Supplementary Figure 3. Phylogenic tree of fucoxanthin-chlorophyll binding proteins.** Each tip of the tree represents a unique amino acid sequence of a putative light harvesting protein. Tips are colored by putative functional group identified by Kumazawa, et al[6] and shaped by if they originated from a reference sequence[6] or from this study. Tree was assembled with IQTree (1,000 bootstrap replicates and bnni option, model VT+R8 chosen according to BIC). Groups (color coded; see key) were determined with a bootstrap cutoff of 60.


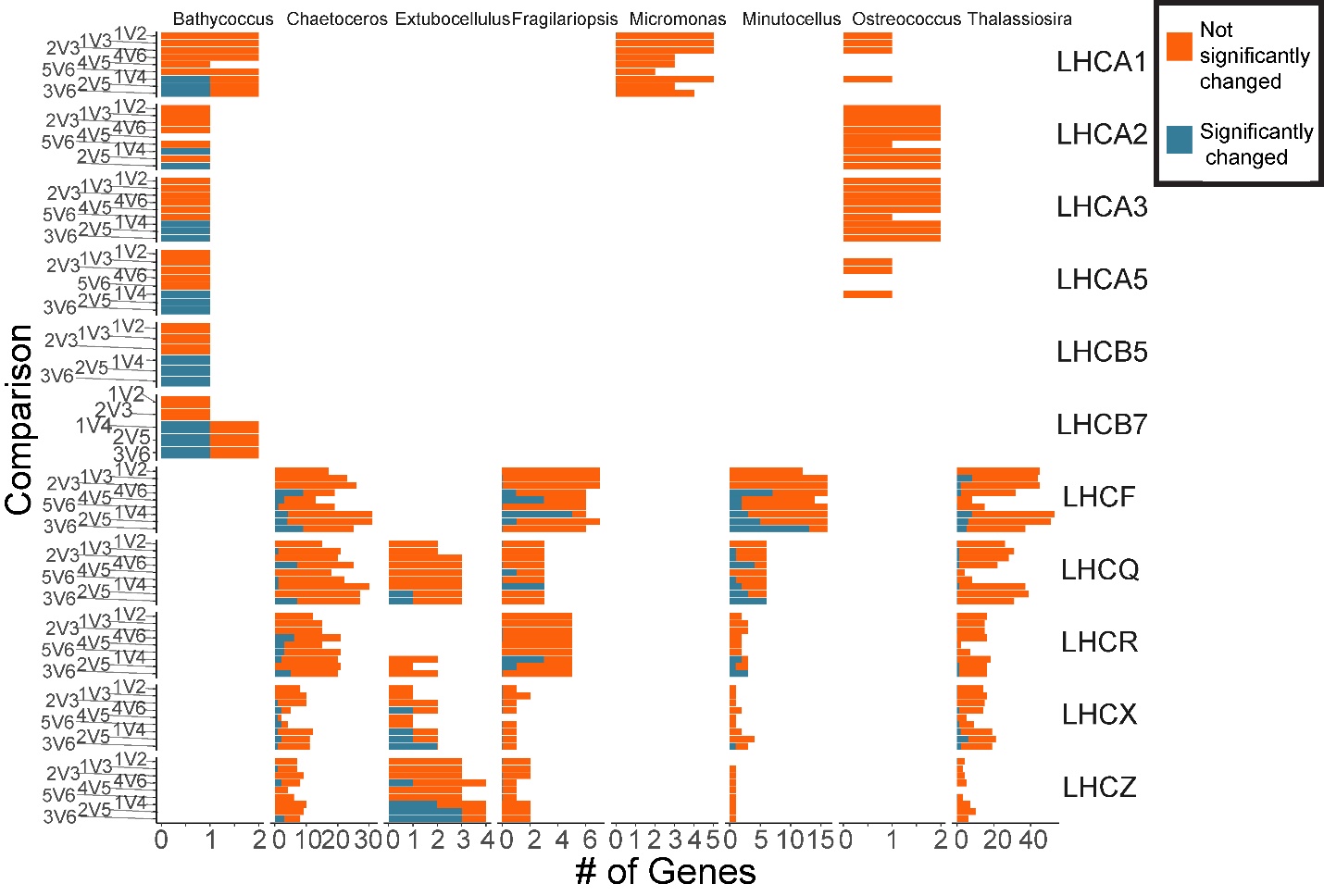
**Supplementary Figure 4. Relative number of photosynthetic LHC genes significantly changed in response to stratification.** Bar plots denoting total transcripts detected and significantly changed in this study. Y-axes refer to the comparisons between water column depths and sampling dates shown in **Figure 2.** “1V2”, 1V3”, “2V3” compare within the deeply mixed water column. “4V6”, “4V5”, “5V6” compare samples within shallow mixed layer water column. “1V4”, “2V5”, “3V6” compare samples at the same relative surface irradiance between the deep mixed layer and shallow mixed layer. Blank spots indicate no genes of that class were detected for that genus. Categories are derived from putative families of photosynthetic LHC genes based on KEGG annotations or Kumazawa et al[6] (indicated on far right). “Significantly changed” (blue bars) refers to genes with an adjusted *p* value <0.001 and fold change >|2|. Columns are arranged by phytoplankton genus.


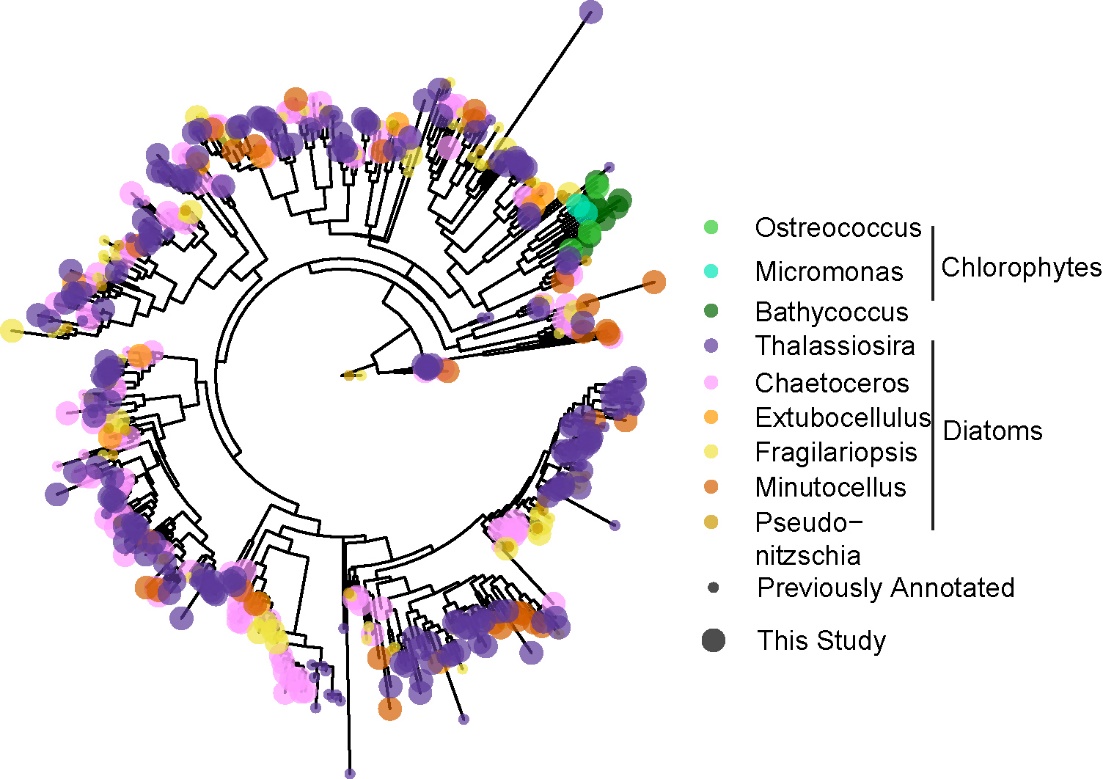


**Supplementary Figure 5. Comparison of Chlorophyte and Diatom Light Harvesting transcript sequences.** Each tip of the tree represents a unique amino acid sequence of a putative light harvesting protein, as identified in **Supplementary Fig. 3** for diatom transcripts or by alignments to KEGG (via EGGNOG and DIAMOND, see methods) for chlorophyte transcripts. Tips of tree are colored by genus and sized by if they originated from a reference sequence [6] or from this study. Tree was assembled with IQTree (1,000 bootstrap replicates and bnni option, model VT+R8 chosen according to BIC).


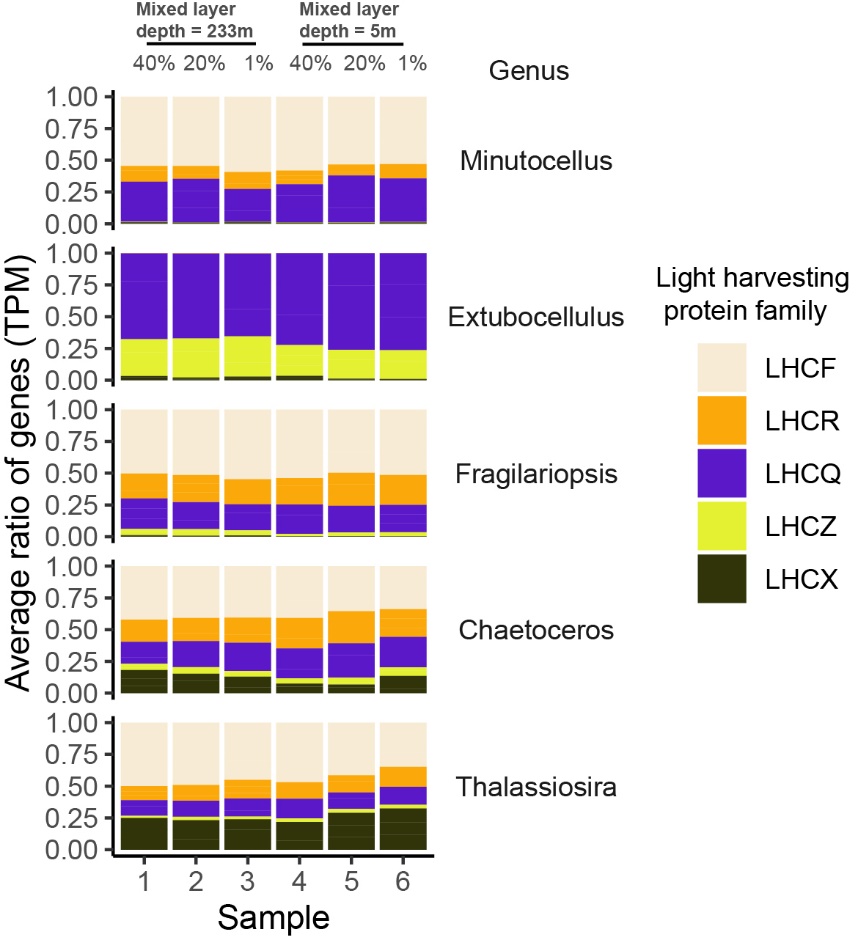
**Supplementary Figure 6. Relative ratios of diatom light harvesting transcripts did not change in response to stratification.** Bar graphs represent the average relative ratio of transcripts per million (TPM, from triplicate measurements) of diatom-specific, photosystem light harvesting protein family genes (colored by type) for different diatom genera (indicated on right). Each columns represents irradiance level relative to the surface (%) within different mixed layer depths (233 m or 5 m). Sampling depths correspond to **Figure 2.**


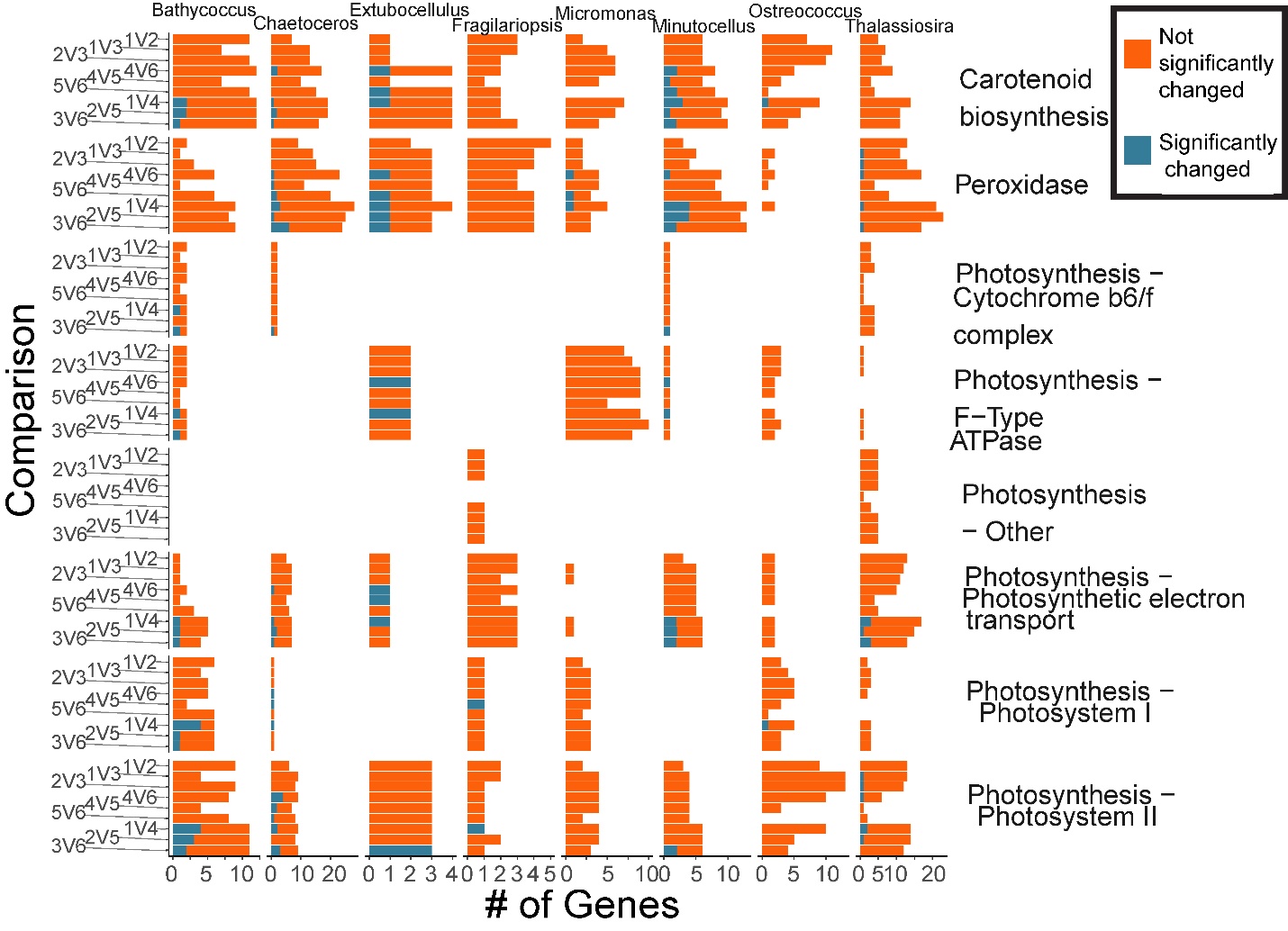
**Supplementary Figure 7. Relative number of photosynthetic and oxidant- scavenging genes significantly changed in response to stratification.** Bar plots denoting total transcripts detected and significantly changed in this study. Y-axes refer to the differential expression comparisons between water column depths and sampling dates shown in **Figure 2.** “1V2”, 1V3”, “2V3” compare within the deeply mixed water column. “4V6”, “4V5”, “5V6” compare samples within shallow mixed layer water column. “1V4”, “2V5”, “3V6” compare samples at the same relative surface irradiance between the deep mixed layer and shallow mixed layer. Blank spots indicate no genes of that class were detected for that genus. Categories are derived from KEGG modules or KEGG pathways (indicated on far right). The category “Significantly changed” (blue bars) refers to genes with an adjusted *p* value <0.001 and fold change >|2|. Columns are arranged by phytoplankton genus.


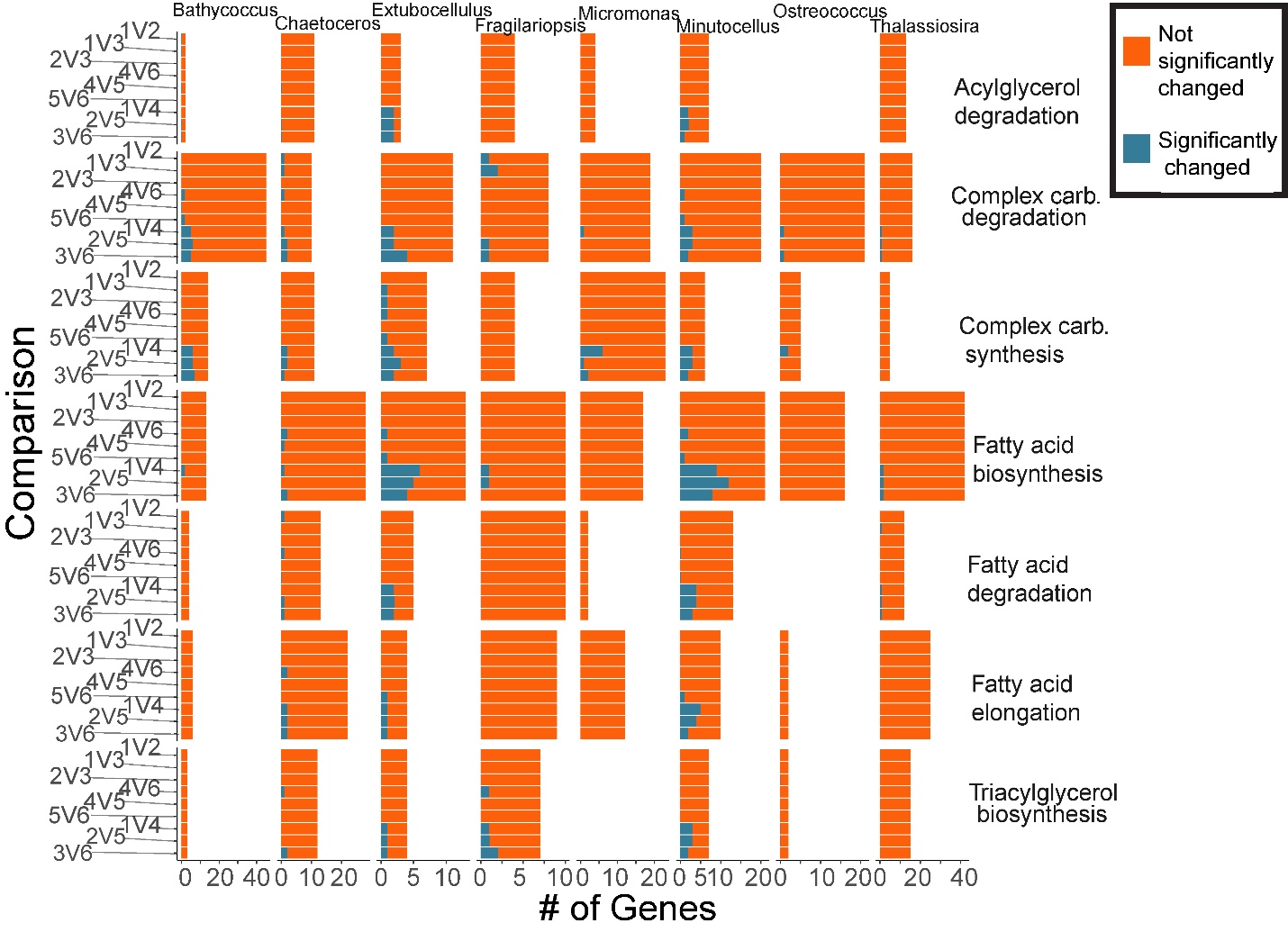


**Supplementary Figure 8. Relative number of energy storage genes significantly changed.** Bar plots denoting total transcripts detected and significantly changed in this study. Y-axes refer to the comparisons between water column depths and sampling dates shown in **Figure 1.** “1V2”, 1V3”, “2V3” compare within the deeply mixed water column. “4V6”, “4V5”, “5V6” compare samples within shallow mixed layer water column. “1V4”, “2V5”, “3V6” compare samples at the same relative surface irradiance between the deep mixed layer and shallow mixed layer. Categories are derived from KEGG modules or KEGG pathways (indicated on far right). “Significantly changed” (blue bars) refers to genes with an adjusted *p* value <0.001 and fold change >|2|. Columns are arranged by phytoplankton genus.


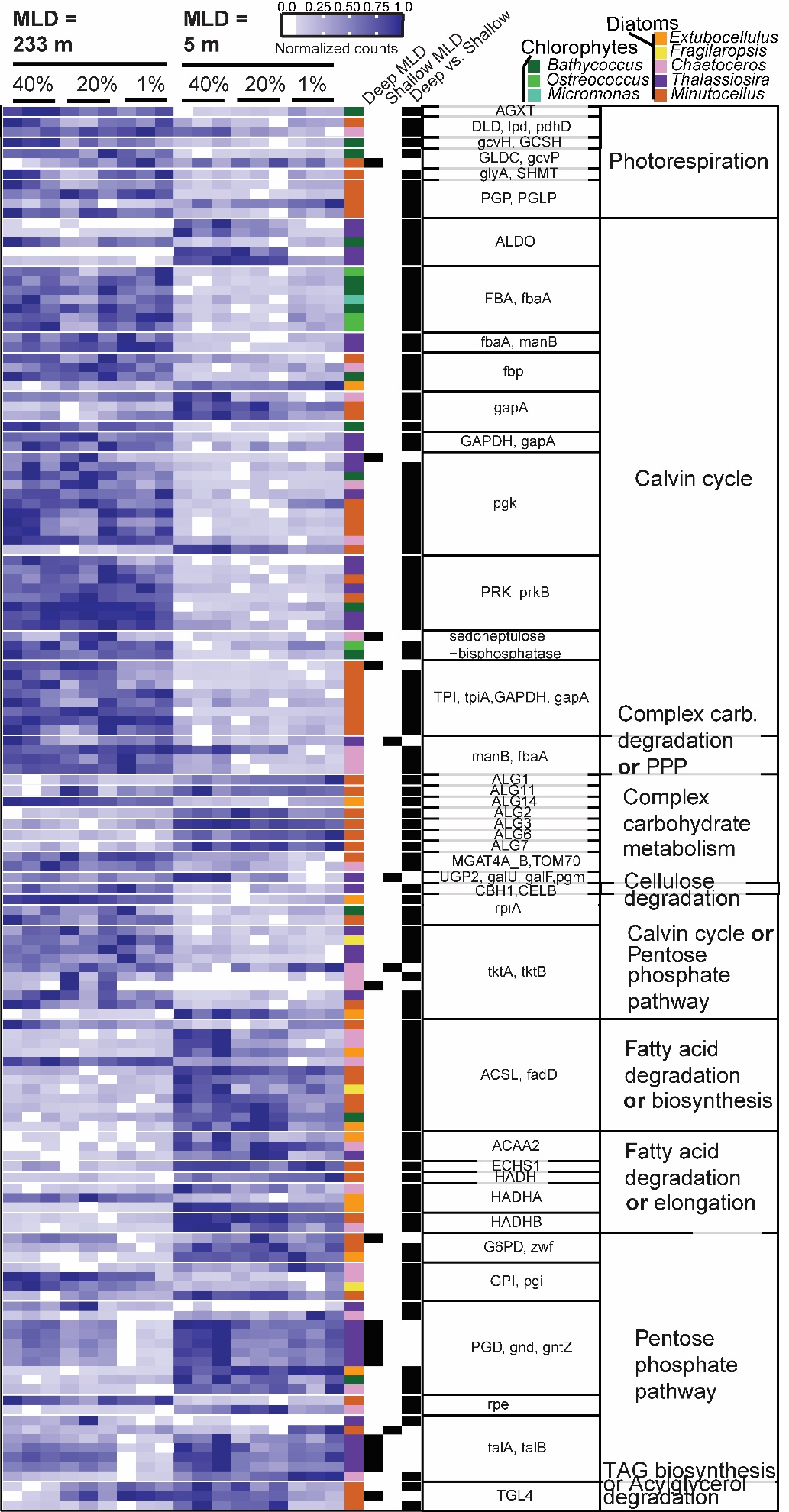


**Supplementary Figure 9. Inverse regulation of carbon fixation (Calvin Cycle) and pentose phosphate pathway genes in response to stratification.** Heatmap of normalized read counts normalized by row to reflect relative expression of each gene (see scale bar). Each row represents a unique transcript (colored by genus; see key) (and each heatmap column represents a triplicate sample from within the euphotic zone in different mixed layer depths (233 m for 24 May and 5 m for 26 May, **Fig. 1A**. The next three columns denote whether each transcript was differentially expressed (black bars) within the water columns from the deeply mixed (“Deep MLD”) or shallow mixed layers (“Shallow MLD”); or between water columns from the same light level (40%, 20% or 1% relative surface irradiance, “Stratified”). Putative gene names are indicated along with module/pathway (on far right). Modules with “or” or “/” (e.g., “Fatty acid degradation or biosynthesis”) did not indicate a clear directionality of the reaction and could be included in two or more of the modules presented in **Fig. 6**.


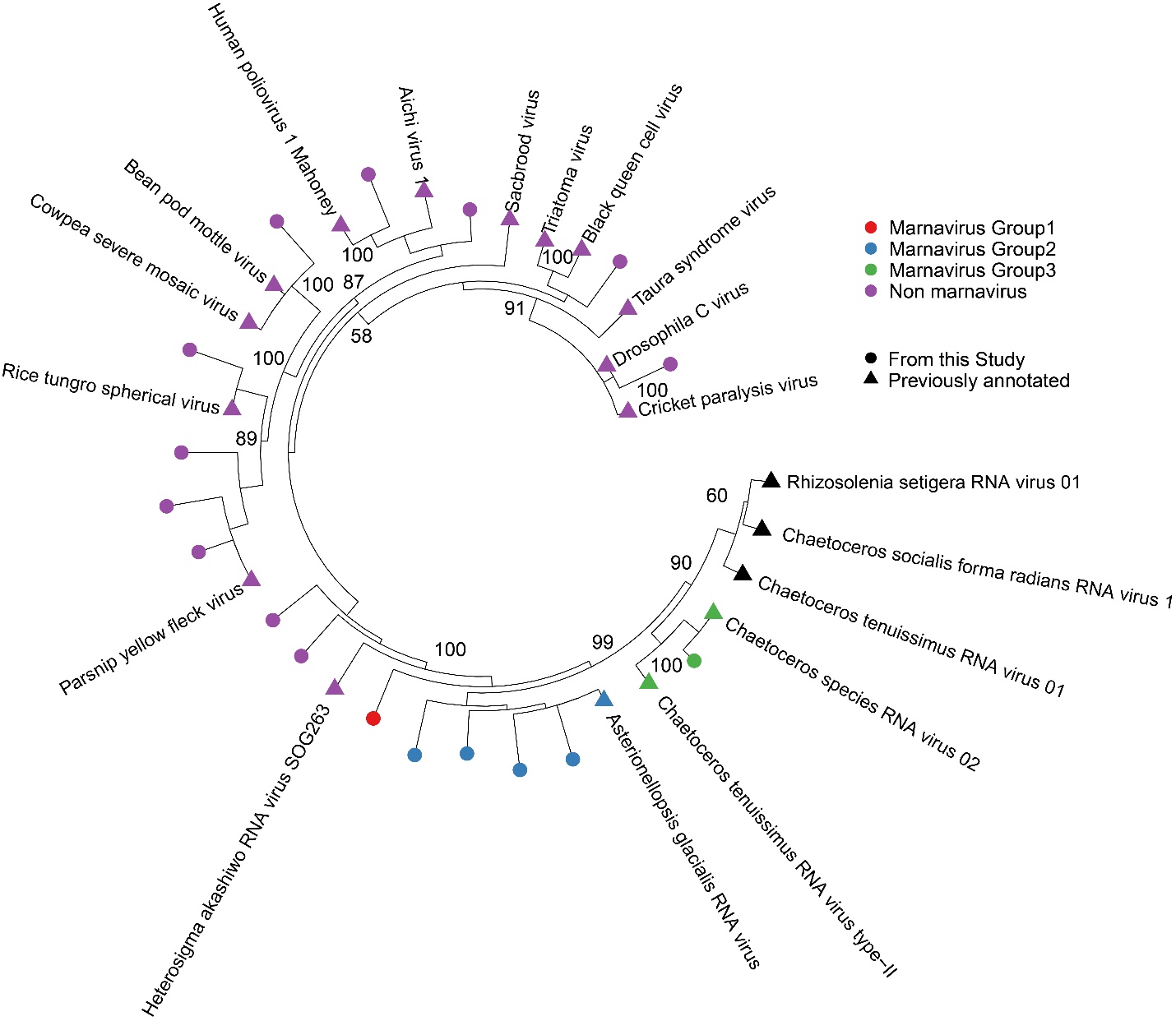


**Supplementary Figure 10. Phylogenic relationships of RNA-dependent RNA polymerase (RdRP) sequences**. Tree was assembled with RaxmL (model = WAG+F+G4, bootstraps = 100) and putative RdRP sequences were placed with PPlacer and GUPPY. Tree tips are shaped by whether they are derived from a reference sequence or from this study. Branch tip shapes indicate if sequences derived from this study or previous studies and are colored depending on if they belong to putative Marnavirus groups (red, blue or green) or not (purple). Non Marnavirus viral transcripts were not used in this study.

**References cited:**

1. Peng X, Fawcett SE, van Oostende N, Wolf MJ, Marconi D, Sigman DM, et al. Nitrogen uptake and nitrification in the subarctic North Atlantic Ocean. Limnol Oceanogr 2018; 63: 1462–1487.

2. Pommier J, Gosselin M, Michel C. Size-fractionated phytoplankton production and biomass during the decline of the northwest Atlantic spring bloom. J Plankton Res 2009; 31: 429–446.

3. Behrenfeld MJ, Moore RH, Hostetler CA, Graff J, Gaube P, Russell LM, et al. The North Atlantic Aerosol and Marine Ecosystem Study (NAAMES): Science motive and mission overview. Front Mar Sci 2019; 6: 122.

4. Diaz BP, Knowles B, Johns CT, Laber CP, Bondoc KG V., Haramaty L, et al. Seasonal mixed layer depth shapes phytoplankton physiology, viral production, and accumulation in the North Atlantic. Nat Commun 2021; 12: 1–16.

5. Della Penna A, Gaube P. Overview of (sub)mesoscale ocean dynamics for the NAAMES field program. Front Mar Sci 2019; 6: 384.

6. Kumazawa M, Nishide H, Nagao R, Inoue‐Kashino N, Shen J, Nakano T, et al. Molecular phylogeny of fucoxanthin-chlorophyll a/c proteins from Chaetoceros gracilis and Lhcq/Lhcf diversity. Physiol Plant 2021; 174: e13598.
